# Integrated Assessment of Phase 2 Data on GalNAc_3_-Conjugated 2′-*O*-Methoxyethyl-Modified Antisense Oligonucleotides

**DOI:** 10.1089/nat.2022.0044

**Published:** 2023-02-01

**Authors:** Brenda F. Baker, Shuting Xia, Wesley Partridge, T. Jesse Kwoh, Sotirios Tsimikas, Sanjay Bhanot, Richard S. Geary

**Affiliations:** ^1^ Department of Drug Development, Ionis Pharmaceuticals, Carlsbad, California, USA.; ^2^ Department of Vascular Medicine, University of California San Diego, La Jolla, California, USA.

**Keywords:** phase 2, integrated safety analysis, randomized placebo-controlled trials, ligand-conjugated antisense technology, triantennary *N*-acetylgalactosamine

## Abstract

Receptor-mediated delivery of an antisense oligonucleotide (ASO) using the ligand-conjugated antisense technology is establishing a new benchmark for antisense therapeutics. The triantennary *N*-acetylgalactosamine (GalNAc_3_) cluster is the first conjugated ligand to yield a marked increase in ASO potency for RNA targets expressed by hepatocytes, compared to the unconjugated form. In this study, we present an integrated safety assessment of data available from randomized, placebo-controlled, phase 2 studies for six GalNAc_3_-conjugated 2′-*O*-methoxyethyl (2′MOE)-modified ASOs. The total study population included 642 participants (130 placebo; 512 ASO) with up to 1 year of exposure. The primary measures were the incidence of signals from standardized laboratory tests and the mean test results over time. The GalNAc_3_-conjugated ASOs were well tolerated with no class effect identified across all doses tested compared to placebo. These results extend prior observations from phase 1 studies, now with treatment up to 1 year.

## Introduction

**FIG. 1.**
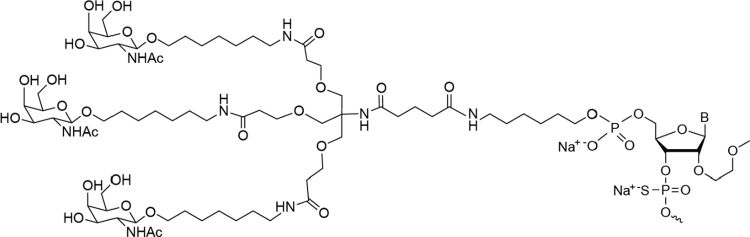
Ligand-conjugated antisense technology. All ASOs are conjugated to the same GalNAc_3_ cluster and linker at the 5′ terminal 2′MOE-modified nucleotide by a phosphodiester group that hydrolyzes after receptor-mediated cellular uptake to release the unconjugated ASO [7,28]. 2′MOE, 2′-*O*-methoxyethyl; ASOs, antisense oligonucleotides; GalNAc_3_, triantennary *N*-acetylgalactosamine.

Nine antisense oligonucleotides (ASOs) have been commercialized to date for indications as diverse as spinal muscular atrophy, hereditary transthyretin amyloidosis, familial chylomicronemia syndrome, and Duchenne muscular dystrophy [1–4]. As the potential of antisense therapeutics is being realized, the technology continues to steadily advance and produce better-performing agents [4]. For example, receptor-mediated delivery of an ASO using the ligand-conjugated antisense technology is one such advancement. Conjugation of a ligand to an ASO allows for productive delivery of the pharmacophore (i.e., the ASO) to the tissue or cellular compartment containing the target RNA. A first-in-class example is the triantennary *N*-acetylgalactosamine (GalNAc_3_) ligand (Fig. 1), designed as a high-affinity ligand for the asialoglycoprotein receptor that is abundantly expressed by hepatocytes [5–10]. The ASO is liberated from the GalNAc_3_ ligand upon receptor-mediated intracellular uptake and released from the endosomal compartment for productive delivery to the target RNA [7].

Sixteen GalNAc_3_-conjugated 2′-*O*-methoxyethyl (2′MOE)-modified ASOs are in clinical development today. Integrated assessment of placebo-controlled phase 1 data on ASOs of this chemical class demonstrated an up to 30-fold increase in potency relative to the unconjugated parent 2′MOE ASO, with no indication of a class effect upon analysis of standard safety laboratory tests across all doses tested in healthy volunteers [2,11–14]. Herein we present the integrated safety assessment of data from seven randomized, placebo-controlled, phase 2 studies for six GalNAc_3_-conjugated 2′MOE ASOs, which were administered monthly or weekly/every other week by subcutaneous injection [15–19].

## Materials and Methods

### Study designs and conduct

The seven clinical trials included in this retrospective integrated data assessment were registered at ClinicalTrials.gov (NCT03385239, NCT03070782, NCT03371355, NCT03020745, NCT03714776, NCT04083222, and NCT04030598). Results from each trial are either published or posted on the registry site [15–19]. All clinical trial protocols were approved by the appropriate institutional review boards and/or independent ethics committees. All studies complied with the Declaration of Helsinki and the International conference on Harmonization Guidelines on Good Clinical Practice. Written and informed consent were obtained from all participants before participation in the study. All studies included a placebo-controlled group. Subcutaneous injection was the route of study drug administration for all study protocols. Evaluable subjects received at least one dose of study drug.

### Safety assessments

Blood samples were collected before dosing for all standard laboratory tests. Urine protein was determined using dipstick. Data were imported from individual study data sets into one SAS dataset for each laboratory test [20,21].

Flu-like reactions (FLRs) were defined as either (a) influenza-like illness or (b) pyrexia or feeling hot or body temperature increased, plus at least two of the following: chills, myalgia, and arthralgia, started on the day of injection or the next day. Local cutaneous reaction at the injection site (LCRIS) was defined as injection site erythema, injection site swelling, injection site pruritus, or injection site pain that started on the day of injection and persisted (start to stop) for 2 days or more.

An alternate calculation, LCRISv2, was defined as moderate or severe injection site erythema, injection site swelling, injection site pruritus, or injection site pain that started on the day of injection and persisted for at least 2 days; or any adverse event (AE) at the injection site, regardless of severity, which leads to discontinuation of study drug, where AE at the injection site is the principal reason for treatment discontinuation. The percentage of injections leading to a tolerability event (FLR, LCRIS, or LCRISv2) was calculated for each subject as follows: (number of injections with event/total number of injections) × 100.

### Statistics

Data assessed in the current integrated analyses were based on an electronic data capture date of March 3, 2021. All data were from completed trials, or from trials that had been unblinded and locked at primary endpoint analysis.

Data were analyzed according to the incidence of events and using descriptive summary statistics of laboratory results. The incidence of events was based on confirmed test results for liver, kidney, hematology, and electrolytes. All study data were included for analysis of the incidence of events. Baseline was defined as the last nonmissing value before the first dose. An event was defined as data falling outside the normal range or reaching the specified threshold, as defined by protocol stopping rules, standard reporting, or an event that meets the criteria established in the Common Terminology Criteria for Adverse Events (CTCAE, version 5.0) [22].

A confirmed event was defined as a consecutive abnormal laboratory value on a different day. If there was no consecutive test to confirm, then the initial observation was presumed confirmed. If there were multiple values on the same day, but sampled at different times, the worst value was used. A persistent event was defined as elevated levels on two consecutive measurements at least 7 days apart, with all values between the initial and subsequent test also meeting the specified threshold. The estimated glomerular filtration rate (eGFR) was calculated using the Chronic Kidney Disease Epidemiology Collaboration equation [23,24].

The over-time analysis of laboratory test results included all study data during the treatment period, which were defined as first dose to 10 days after the last dose for subjects dosed weekly, 17 days after the last dose for those dosed every other week, and 31 days after the last dose for those dosed monthly.

A meta-analysis using subject-level data was performed to compare ASO-treated dose groups with the placebo group. The endpoints evaluated were the absolute changes from baseline. Data were compared between GalNAc_3_-conjugated 2′MOE ASO dose groups and placebo using an analysis of covariance model, with the dose group and trial as fixed factors and baseline value as a covariate. Two-sided *P* values were reported. Because of the exploratory nature of this analysis, *P* values were not adjusted for multiplicity.

Statistical analyses were performed using SAS version 9.3.

## Results

### Integrated study population

Data were obtained from seven randomized, placebo-controlled, dose ranging, phase 2 trials for six GalNAc_3_-conjugated ASOs. The total clinical study population consisted of 642 subjects who were assigned to a multiple dose regimen and received at least one dose of study drug (placebo: *n* = 130, ASO: *n* = 512; Supplementary Table S1). Patient demographics, baseline laboratory tests, concomitant medications, and medical histories were similar between the placebo and ASO groups (Tables 1 and 2). Two dose regimen cohorts were also analyzed in this assessment (Supplementary Table S2) and included those who received monthly dosing (referred to as the monthly dose regimen cohort) and those who received dosing every other week or weekly (referred to as the weekly dose regimen cohort).

**Table 1.** Baseline Characteristics of the Total Study Population

Parameter	Placebo	Total ASO	Dose (mg/month)				
			>0 to <40	40 to <80	80 to <160	160 to <320	≥320
*N*	130	512	93	214	120	35	50
Age							
Mean (SD), years	57.3 (11.1)	58.3 (11.1)	63.2 (9.6)	60.2 (10.7)	54.5 (10.9)	47.5 (9.6)	57.1 (9.2)
Sex							
Male, *n* (%)	75 (57.7%)	328 (64.1%)	62 (66.7%)	150 (70.1%)	66 (55.0%)	23 (65.7%)	27 (54.0%)
Race							
White, *n* (%)	107 (82.3%)	427 (83.4%)	88 (94.6%)	204 (95.3%)	110 (91.7%)	0	25 (50.0%)
Asian, *n* (%)	13 (10.0%)	60 (11.7%)	1 (1.1%)	1 (0.5%)	7 (5.8%)	35 (100%)	16 (32.0%)
Black, *n* (%)	9 (6.9%)	21 (4.1%)	3 (3.2%)	7 (3.3%)	3 (2.5%)	0	8 (16.0%)
Other, *n* (%)	1 (0.8%)	4 (0.8%)	1 (1.1%)	2 (0.9%)	0	0	1 (2.0%)
Body mass index							
Mean (SD), kg/m^2^	28.8 (4.5)	29.1 (4.9)	30.4 (4.4)	29.6 (4.5)	30.0 (5.4)	23.2 (2.6)	26.7 (4.2)
Alanine transaminase							
Mean (SD), U/L	27.4 (16.5)	23.4 (10.9)	22.4 (10.3)	25.0 (11.3)	24.8 (11.4)	18.8 (7.7)	18.5 (8.2)
>ULN, *n* (%)	15 (11.5%)	35 (6.8%)	5 (5.4%)	17 (7.9%)	11 (9.2%)	1 (2.9%)	1 (2%)
Serum creatinine							
Mean (SD), mg/dL	0.82 (0.19)	0.84 (0.19)	0.88 (0.18)	0.87 (0.18)	0.80 (0.20)	0.75 (0.15)	0.81 (0.20)
>ULN, *n* (%)	0 (0%)	1 (0.2%)	0 (0%)	0 (0%)	1 (0.8%)	0 (0%)	0 (0%)
eGFR (CKD-EPI)							
Mean (SD), mL/min per 1.73 m^2^	92.0 (15.8)	90.7 (16.1)	85.1 (14.3)	87.9 (15.6)	94.6 (17.1)	105.8 (9.4)	93.1 (14.4)
<90 mL/min per 1.73 m^2^, *n* (%)	57 (43.8%)	226 (44.1%)	53 (57%)	116 (54.2%)	38 (31.7%)	1 (2.9%)	18 (44.1%)
<60 mL/min per 1.73 m^2^, *n* (%)	3 (2.3%)	11 (2.1%)	5 (5.4%)	3 (1.4%)	3 (2.5%)	0 (0%)	0 (0%)
Platelets							
Mean (SD), K/μL	234.8 (69.0)	231.3 (59.3)	229.7 (61.9)	224.8 (57.1)	235.5 (57.1)	236.9 (60.5)	248.4 (65.8)
<LLN, *n* (%)	5 (3.8%)	23 (4.5%)	2 (2.2%)	12 (5.6%)	7 (5.8%)	0 (0.0%)	2 (4%)

ASO, antisense oligonucleotide; CKD-EPI, Chronic Kidney Disease Epidemiology Collaboration (calculation); eGFR, estimated glomerular filtration rate; LLN, lower limit of normal; SD, standard deviation; ULN, upper limit of normal.

**Table 2.** Medical History and Concomitant Medications for the Total Study Population

Incidence,* n *(%)	Placebo	Total ASO	Dose (mg/month)				
			>0 to <40	40 to <80	80 to <160	160 to <320	≥320
*N*	130	512	93	214	120	35	50
**Medical history**							
Hypertension	103 (79.2%)	434 (84.8%)	92 (98.9%)	203 (94.9%)	90 (75%)	7 (20%)	42 (84%)
Hypertriglyceridemia	98 (75.4%)	407 (79.5%)	93 (100%)	203 (94.9%)	83 (69.2%)	2 (5.7%)	26 (52%)
Cardiovascular disorders	72 (55.4%)	340 (66.4%)	93 (100%)	190 (88.8%)	54 (45%)	0 (0%)	3 (6%)
Diabetes	61 (46.9%)	197 (38%)	44 (47.3%)	74 (34.6%)	58 (48.3%)	2 (5.7%)	19 (38%)
Hepatobiliary disorders	39 (30%)	134 (26.2%)	3 (3.2%)	39 (18.2%)	57 (47.5%)	22 (62.9%)	13 (26%)
Renal impairment	8 (6.2%)	50 (9.8%)	14 (15.1%)	22 (10.3%)	10 (8.3%)	1 (2.9%)	3 (6%)
**Concomitant medications**							
Lipid-modifying agents	87 (66.9%)	389 (76%)	89 (95.7%)	195 (91.1%)	81 (67.5%)	3 (8.6%)	21 (42.0%)
HMG CoA reductase inhibitors	81 (62.3%)	355 (69.3%)	79 (84.9%)	180 (84.1%)	74 (61.7%)	2 (5.7%)	20 (40.0%)
Ezetimibe	27 (20.8%)	126 (24.6%)	28 (30.1%)	70 (32.7%)	26 (21.7%)	1 (2.9%)	1 (2%)
Alirocumab or evolocumab	11 (8.5%)	60 (11.7%)	13 (14.0%)	34 (15.9%)	13 (10.8%)	0 (0%)	0 (0%)
Antithrombotic agents	69 (53.1%)	343 (67.0%)	87 (93.5%)	186 (86.9%)	59 (49.2%)	2 (5.7%)	9 (18%)
Agents acting on renin-angiotensin system	71 (54.6%)	279 (54.5%)	63 (67.7%)	127 (59.3%)	58 (48.3%)	3 (8.6%)	28 (56%)
Beta-blocking agents	48 (36.9%)	234 (45.7%)	64 (68.8%)	122 (57.0%)	41 (34.2%)	0 (0%)	7 (14.0%)
Drugs used in diabetes	58 (44.6%)	179 (35.0%)	38 (40.9%)	71 (33.2%)	58 (48.3%)	0 (0%)	12 (24%)
Diuretics	25 (19.2%)	99 (19.3%)	23 (24.7%)	44 (20.6%)	22 (18.3%)	0 (0%)	10 (20.0%)
Calcium channel blockers	22 (16.9%)	89 (17.4%)	19 (20.4%)	40 (18.7%)	12 (10.0%)	5 (14.3%)	13 (26.0%)

HMG CoA, 3-hydroxy-3-methylglutaryl coenzyme-A.

The monthly dose regimen cohort consisted of 318 subjects (placebo, *n* = 65; ASO, *n* = 253) and the weekly dose regimen cohort consisted of 324 subjects (placebo, *n* = 65; ASO, *n* = 259). The demographics and baseline values of alanine transaminase (ALT), serum creatinine, calculated GFR, and platelets for each dose regimen cohort are summarized in Supplementary Tables S3 and S4.

### Standard clinical laboratory tests

#### Liver

Liver parameters included ALT, aspartate transaminase (AST), albumin, alkaline phosphatase, total bilirubin, and Hy's Law (Table 3). No dose-dependent signals were identified across these liver parameters. One of 509 ASO-treated subjects (0.2%) had a confirmed ALT increase >5 × upper limit of normal (ULN) and concomitant confirmed AST increase >5 × ULN. This increase was not associated with an increase in total bilirubin and occurred during the post-treatment period. The subject was assigned to the lowest dose category (>0 to 40 mg/month) of the monthly dose regimen (Supplementary Table S5). Two other subjects in higher dose categories (80 to <160 and 160 to <320 mg/month) experienced confirmed ALT elevations >3 × ULN (Table 3); both were from the weekly dose regimen cohort (Supplementary Table S6).

**Table 3.** Incidence of Abnormal Laboratory Tests in the Total Study Population

Incidence of events^a^	Placebo	Total ASO	Dose category (mg/month)				
			>0 to <40	40 to <80	80 to <160	160 to <320	≥320
**Liver**							
ALT,^b^ *n*	129	509	91	214	120	35	49
>3 × ULN, or BL if >ULN	0	3 (0.6%)	1 (1.1%)	0	1 (0.8%)	1 (2.9%)	0
>5 × ULN, or BL if >ULN	0	1 (0.2%)	1 (1.1%)	0	0	0	0
AST,^b^ *n*	129	509	91	214	120	35	49
>3 × ULN, or BL if >ULN	0	1 (0.2%)	1 (1.1%)	0	0	0	0
>5 × ULN, or BL if >ULN	0	1 (0.2%)	1 (1.1%)	0	0	0	0
Albumin, *n*	129	509	91	214	120	35	49
<LLN, or BL if <LLN	0	1 (0.2%)	0	1 (0.5%)	0	0	0
<2.5 g/dL	0	0	0	0	0	0	0
Alkaline phosphatase, *n*	129	509	91	214	120	35	49
>3 × ULN, or BL if >ULN	0	0	0	0	0	0	0
Total bilirubin, *n*	129	509	91	214	120	35	49
>2 × ULN, or BL if >ULN	0	0	0	0	0	0	0
Hy's law^c^	129	509	91	214	120	35	49
Total bilirubin >2 × ULN and ALT >3 × ULN, or BL if >ULN	0	0	0	0	0	0	0
**Kidney**							
Serum creatinine, *n*	129	509	91	214	120	35	49
≥0.3 mg/dL inc., from BL, or ≥1.5 × BL	3 (2.3%)	12 (2.3%)	4 (4.3%)	2 (0.9%)	6 (5.0%)	0	0
≥2 × BL	0	1 (0.2%)	0	0	1 (0.8%)	0	0
>2.1 mg/dL	0	0	0	0	0	0	0
Blood urea nitrogen, *n*	129	509	91	214	120	35	49
≥2 × ULN, or BL if >ULN	0	0	0	0	0	0	0
eGFR CKD-EPI, *n*	129	509	91	214	120	35	49
<60 mL/min per 1.73 m^2^	9 (6.9%)	36 (7.0%)	11 (11.8%)	16 (7.5%)	8 (6.7%)	0	1 (2.0%)
<30 mL/min per 1.73 m^2^	0	0	0	0	0	0	0
Urine protein, *n*	129	509	91	214	120	35	49
≥2+ (100 mg/dL)	4 (3.1%)	11 (2.1%)	3 (3.2%)	5 (2.3%)	2 (1.7%)	1 (2.9%)	0
≥3+ (200 mg/dL)	0	0	0	0	0	0	0
**Hematology**							
Platelets, *n*	129	509	91	214	120	35	49
<75 K/μL	0	0	0	0	0	0	0
<50 K/μL	0	0	0	0	0	0	0
Hemoglobin, *n*	129	508	91	214	120	34	49
Males <10.5 g/dL; females <9.5 g/dL	0	3 (0.6%)	1 (1.1%)	1 (0.5%)	1 (0.8%)	0	0
Hematocrit, *n*	129	508	91	214	120	34	49
<0.85 × BL	2 (1.5%)	13 (2.5%)	7 (7.5%)	3 (1.4%)	1 (0.8%)	0	2 (4.0%)
<30% (Absolute value)	0	0	0	0	0	0	0
Lymphocytes, *n*	127	497	91	214	119	28	45
<0.5 K/μL	0^1^	0	0	0	0	0	0
Absolute neutrophil count, *n*	129	505	91	214	119	33	48
<1.0 K/μL	0	0	0	0	0	0	0
**Serum electrolytes**							
Potassium, *n*	129	509	91	214	120	35	49
<3.0 mmol/L	0	0	0	0	0	0	0
>5.5 mmol/L	0	5 (1.0%)	0	3 (1.4%)	1 (0.8%)	0	1 (2.0%)
Sodium, *n*	129	509	91	214	120	35	49
<130 mmol/L	0	6 (1.2%)	1 (1.1%)	1 (0.5%)	1 (0.8%)	0	3 (6.0%)
>150 mmol/L	0	0	0	0	0	0	0
Bicarbonate, *n*	119	453	91	214	114	0	34
<LLN, or BL if <LLN	2 (1.5%)	9 (1.8%)	2 (2.2%)	7 (3.3%)	0	0	0
Chloride, *n*	119	453	91	214	114	0	34
>ULN, or BL if >ULN	2 (1.5%)	0	0	0	0	0	0

^a^ Results shown are confirmed events, defined as a consecutive abnormal laboratory value on next measurement after the initial observation and on a different day, unless specified otherwise. If there is no consecutive test to confirm, the initial observation is presumed confirmed.

^b^ Elevated levels on two consecutive measurements at least 7 days apart with all values between the initial and subsequent test also above (or below) the specified threshold.

^c^ ALT and total bilirubin must meet the criteria on the same day.

BL, baseline; ALT, alanine transaminase; AST, aspartate transaminase.

**FIG. 2.**
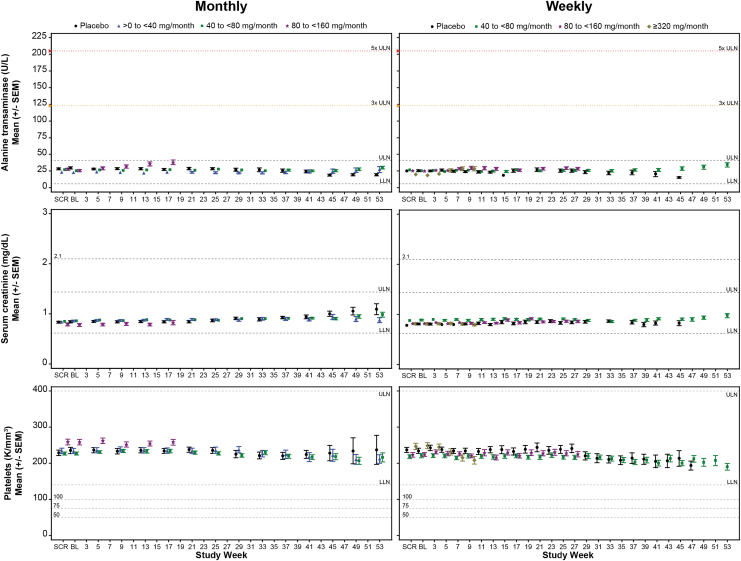
Mean sentinel laboratory tests over time by dose regime cohort, monthly (*left column*) and weekly (*right column*). Sentinel laboratory tests are for liver, alanine transaminase; kidney, serum creatinine; and hematology, platelets. The LLN and ULN displayed represent the median values. Screening was defined as average of all values measured before baseline. Each data point represents at least 6 subjects and 2 ASOs. LLN, lower limit of normal; SEM, standard error of the mean; ULN, upper limit of normal.

No marked effect of GalNAc-conjugated ASO treatment on ALT levels was observed over time by dose category, with mean values remaining within the range of normal during the treatment period for both monthly and weekly dose regimens (Fig. 2). Differences in the least-squares mean between ASO dose categories and placebo were statistically significant at the highest dose categories at week 5 and persisted to last measurement of the treatment period in both the monthly and weekly dose regimes (80 to <160 mg/month and ≥320 mg/month, respectively) (Supplementary Tables 7 and 8). Changes in ALT were accompanied by changes in AST, but not in albumin, alkaline phosphatase, or total bilirubin (Supplementary Fig. S1; Supplementary Tables S9 and S10).

#### Kidney

Measurements of renal function included serum creatinine, blood urea nitrogen (BUN), eGFR, and urine protein (Table 3). No dose-dependent safety signal was identified across kidney tests. Serum creatinine was elevated to ≥0.3 mg/dL above or ≥1.5 × baseline in 3 (2.3%) subjects receiving placebo and 12 (2.3%) subjects receiving ASO (Table 3); 6 (2.4%) of the ASO-treated subjects were from the monthly dose regimen cohort (Supplementary Table S5) and 6 (2.3%) were from the weekly dose regimen cohort (Supplementary Table S6). One of the six subjects from the weekly dose regimen cohort also reached serum creatinine levels >2 × baseline.

There was no marked effect of GalNAc_3_-conjugated ASO treatment on kidney function over time by dose category. Mean values of serum creatinine remained within the range of normal for both monthly and weekly dose regimens (Fig. 2). The serum creatinine mean change from baseline exhibited occasional statistical difference compared to placebo, but these changes were transient with no observable trend (Supplementary Tables S7 and S8). Mean values over time in BUN remained within the range of normal and mean eGFR remained above 90 mL/min per 1.73 m^2^ (Supplementary Fig. S2). Differences in the mean changes from baseline between placebo and ASO dose categories over time for BUN and eGFR were overall unremarkable (Supplementary Tables S11 and S12).

#### Hematology

Hematological measurements included platelets, hemoglobin, hematocrit, lymphocyte count, and absolute neutrophil count. There was no case in either treatment group, placebo or total ASO, of subjects experiencing a platelet count below 75 K/μL on study (Table 3). Although both hemoglobin and hematocrit showed a higher incidence of abnormal events in the total ASO group, no dose dependence was observed. There were no cases of reduced counts in lymphocytes or absolute neutrophils below the specified thresholds of 0.5 and 1.0 K/μL, respectively.

Mean platelet counts over time remained within the range of normal across all dose categories (Fig. 2). No significant difference was observed in the mean change from baseline between placebo and ASO dose categories in the monthly dose regimen cohort (Supplementary Table S7). However, a significant difference in the mean change from baseline was observed between placebo and the highest dose category (≥320 mg/month) in the weekly dose regimen cohort at week 5; this difference persisted through week 9 (Supplementary Table S8) and is attributed to a single sequence and study population. Although this finding is limited by sample size and number of ASOs, previous integrated assessments of the unconjugated form of the 2′MOE ASOs found a small proportion of sequences displayed a dose-dependent effect on platelet levels based on percent reduction from baseline rather than clinically significant events [25].

The mean values over time for other parameters remained within the range of normal over time (Supplementary Fig. S3), with no significant difference in mean changes from baseline between placebo and ASO dose categories (Supplementary Tables S13 and S14).

#### Serum electrolytes

Electrolyte measurements included serum potassium, sodium, bicarbonate, and chloride. In the total study population, there was no reduction to <3.0 mmol/L, and 5 of 509 (1.0%) ASO-treated subjects experienced increased potassium levels >5.5 mmol/L (Table 3). Inversely, 6 (1.2%) ASO-treated subjects experienced reductions in sodium <130 mmol/L with no case of sodium >150 mmol/L. Sodium decreases demonstrated some dose dependence, with 3 (6.0%) subjects in the highest dose group (≥320 mg/month) experiencing reductions to <130 mmol/L. Five of the six subjects who experienced a reduced serum sodium level were from the weekly dose regimen cohort (Supplementary Tables S5 and S6). Bicarbonate was decreased below the lower limit of normal equally in the placebo- (*n* = 2 [1.5%]) and ASO-treated (*n* = 9 [1.8%]) subjects, while chloride was only increased above the ULN in the placebo-treated group.

There was no effect of GalNAc_3_-conjugated ASO treatment on serum electrolytes over time by dose category, with all means remaining within the range of normal for both monthly and weekly dose regimens (Supplementary Fig. S4). Sodium showed significant differences in the mean change from baseline between placebo and ASO dose categories, but these observations were transient and independent of dose (Supplementary Tables S15 and S16). These small differences likely reflect the variance from a proportion of patients in the study population on concomitant medications that effect kidney function, for example, diuretics and agents acting on renin-angiotensin system (Table 2), which could perturb serum electrolyte homeostasis.

### Tolerability of GalNAc_3_-conjugated ASOs

Treatment with GalNAc_3_-conjugated ASOs was well tolerated overall. AEs resulted in dose discontinuations for 3 of 130 (2.3%) subjects in the placebo group and for 23 of 512 (4.5%) subjects receiving ASOs; no dose-dependent or sequence-specific relationship was observed in AEs leading to discontinuation (Supplementary Table S17). LCRIS occurred in 65 (12.7%) subjects receiving ASOs. Overall, no dose-dependent or sequence-specific relationship was observed in the incidence of LCRIS. The weekly dose regimen, however, tended to show a higher incidence than the monthly dose regimen cohort (Supplementary Table S18).

The mean percentage of injections leading to an LCRIS event was 2.2% with a median of 0%. The incidence of moderate or severe LCRIS, or LCRIS of any severity leading to discontinuation occurred in 8 (1.6%) of all ASO-treated subjects with a mean percentage of injections of 0.1% (Supplementary Table S18). FLRs occurred in 7 (1.4%) of subjects receiving an ASO, and the mean percentage of injections leading to an event was 0.3%, with a median of 0%. There was no incidence of FLRs in the placebo-treated group.

## Discussion

This study summarizes the phase 2 experience of six GalNAc_3_-conjugated 2′MOE-modified ASOs in 512 participants with up to 1 year of exposure. The GalNAc_3_-conjugated ASOs were well tolerated with no class effect identified across all monthly doses tested compared with placebo. These results extend prior observations from phase 1 studies, now with treatment up to 1 year. Conjugation of the GalNAc_3_ moiety is the first example of targeted delivery of 2′MOE ASOs in humans and represents a significant advance for RNA targets expressed in hepatocytes.

The improved safety margin observed is driven by increased potency, which allows lower doses to achieve full activity and less frequent administration. When comparing monthly dosing to more frequent administration, the safety profile is further improved. Monthly dosing showed no effect on mean values over time in ALT levels, serum creatinine levels, or platelet counts across all dose levels tested over a 6- to 12-month treatment period when compared with placebo administration.

The most remarkable improvement in less frequent dosing (monthly) was demonstrated by the tolerability profile and near-complete reduction of discontinuations due to AEs. The incidence of discontinuing dosing due to an AE was similar between the total ASO (3.6%) and placebo (4.6%) groups of subjects treated under the monthly dose regimen, with <1% of discontinuations attributed to a local AE at the injection site in ASO-treated subjects. The incidence of LCRIS and related dose discontinuations are reduced in comparison to the unconjugated form of the 2′MOE-modified ASOs [26]. This improvement is attributed to subcutaneous (SC) administration of lower doses and the reduction in local concentration of drug at the SC injection site [27].

The expansion of safety and tolerability beyond Phase 1 experience in healthy volunteers provides strength to the current body of evidence. Further strength is provided by the inclusion of controlled data from placebo administration in subjects, the number of drugs in this chemical class (of differing sequences, but similar chemistry), and substantial increase in the duration of exposure up to 1 year. In addition to similar chemistry, the GalNAc_3_ ligand and linker chemistry are identical for all six conjugated ASOs, broadly enabling this chemistry. The primary limitations of this dataset are the relatively small size of the control group and exposures that differ across a broad dose range, such that the highest doses have fewer exposed subjects.

The six GalNAc_3_-conjugated ASOs were well tolerated, with no class effect identified from the integrated laboratory test results compared with placebo in this integrated safety data analysis, the largest phase 2 assessment available to date. These results extend prior observations from phase 1 studies with dosing intervals up to once a month and treatment up to 1 year. This clinical safety profile supports advanced development and the potential of this targeting approach to usher in additional medicines beyond the nine market approvals thus far achieved [1]. Indeed, of the six GalNAc_3_-conjugated 2′MOE ASOs represented in this clinical portfolio, three have now entered final registration, enabling Phase 3 development.

## Supplementary Material

Supplemental data

Supplemental data

Supplemental data

Supplemental data

Supplemental data

Supplemental data

Supplemental data

Supplemental data

Supplemental data

Supplemental data

Supplemental data

Supplemental data

Supplemental data

Supplemental data

Supplemental data

Supplemental data

Supplemental data

Supplemental data

Supplemental data

Supplemental data

Supplemental data

Supplemental data
