# Supplementary material for: Integrated Assessment of Phase 2 Data on GalNAc3-Conjugated 2′-O-Methoxyethyl-Modified Antisense Oligonucleotides
Source: Nucleic Acid Ther. 2023 Feb 1;33(1):72–80. doi: 10.1089/nat.2022.0044 (PMC10623620; doi:10.1089/nat.2022.0044)
Supplement: Supplemental data [file Suppl_TableS1.pdf]

Supplemental Table 1: $GalNAc_3$-conjugated ASOs in Phase 2 Assessment

IONIS-L_{Rx}	Sequence (5'3')	Indication	Placebo, n	ASO, n	Total, N
ApoCIII-L	AGCTTCTTGTCCAGCTTTAT	FCS/Dyslipidemia	24	90	114
Apo(a)-L	TGCTCCGTTGGTGCTTGTTC	Coronary Artery Disease	47	239	286
ANGPTL3-L	GGACATTGCCAGTAATCGCA	Dyslipidemia	27	78	105
HBV-L	GCAGAGGTGAAGCGAAGTGC	HBV Infection	10	56	66
AGT-L	CACAAACAAGCTGGTCGGTT	Resistant Hypertension	16	35	51
PKK-L	TGCAAGTCTCTTGGCAAACA	Hereditary Angioedema	6	14	20
**TOTAL**			**130**	**512**	**642**

ASO, antisense oligonucleotide.
