# Supplementary material for: Integrated Assessment of Phase 2 Data on GalNAc3-Conjugated 2′-O-Methoxyethyl-Modified Antisense Oligonucleotides
Source: Nucleic Acid Ther. 2023 Feb 1;33(1):72–80. doi: 10.1089/nat.2022.0044 (PMC10623620; doi:10.1089/nat.2022.0044)
Supplement: Supplemental data [file Suppl_TableS2.pdf]

Supplemental Table 2. Monthly and weekly dose regimen cohorts

Dose Regimen	IONIS-L_{Rx}	Placebo, n	ASO, n	Total, N
**Monthly (Q4W)**	ApoCIII-L	12	44	56
	Apo(a)-L	29	143	172
	ANGPTL3-L	18	52	70
	PKK-L	6	14	20
	**Total**	**65**	**253**	**318**
**Weekly (QW) / (Q2W)**	ApoCIII-L	12	46	58
	Apo(a)-L	18	96	114
	ANGPTL3-L	9	26	35
	HBV-L	10	56	66
	AGT-L	16	35	51
	**TOTAL**	**65**	**259**	**324**
