# Supplementary material for: Integrated Assessment of Phase 2 Data on GalNAc3-Conjugated 2′-O-Methoxyethyl-Modified Antisense Oligonucleotides
Source: Nucleic Acid Ther. 2023 Feb 1;33(1):72–80. doi: 10.1089/nat.2022.0044 (PMC10623620; doi:10.1089/nat.2022.0044)
Supplement: Supplemental data [file Suppl_TableS3.pdf]

Supplemental Table 3. Baseline characteristics of patients in the monthly dose regime cohort

			Dose (mg/month)		
**Parameter**	**Placebo**	**Total ASO**	**>0 to <40**	**40 to <80**	**80 to <160**
**N**	65	253	70	143	40
**Age, Mean (SD)**	55.5 (12.2)	58.7 (11.5)	61.4 (9.6)	60.2 (10.5)	48.6 (12.9)
**Male, n (%)**	42 (64.6%)	162 (64.0%)	43 (61.4%)	101 (70.6%)	18 (45.0%)
**Race, n (%)**					
White	60 (92.3%)	237 (93.7%)	65 (92.9%)	134 (93.7%)	38 (95.0%)
Black	2 (3.1%)	11 (4.3%)	3 (4.3%)	7 (4.9%)	1 (2.5%)
Asian	3 (4.6%)	3 (1.2%)	1 (1.4%)	1 (0.7%)	1 (2.5%)
Other	0	2 (0.8%)	1 (1.4%)	1 (0.7%)	0
**BMI (kg/m^2), Mean (SD)**	28.7 (3.9)	29.9 (4.7)	30.1 (4.6)	29.5 (4.5)	31.2 (5.5)
**Alanine Transaminase**					
Mean (SD), U/L	29.5 (16.7)	24.4 (11.7)	22.5 (11.1)	25.1 (11.3)	25.5 (13.4)
>ULN, n (%)	8 (12.3%)	22 (8.7%)	5 (7.1%)	10 (7%)	7 (17.5%)
**Serum Creatinine**					
Mean (SD), mg/dL	0.84 (0.20)	0.85 (0.19)	0.86 (0.19)	0.86 (0.17)	0.78 (0.22)
>ULN, n (%)	0 (0%)	1 (0.3%)	0 (0%)	0 (0%)	1 (2.5%)
**eGFR (CKD-EPI)**					
Mean (SD), mL/min/1.73 m^2	92.6 (16.7)	90.2 (15.9)	87.6 (14.2)	88.9 (15.2)	99.2 (18.1)
< 90 mL/min/1.73 m^2, n (%)	29 (44.6%)	117 (46.2%)	34 (48.6%)	75 (52.4%)	8 (20%)
< 60 mL/min/1.73 m^2, n (%)	2 (3.1%)	6 (2.4%)	4 (5.7%)	1 (0.7%)	1 (2.5%)
**Platelets**					
Mean (SD), k/uL	235.3 (74.5)	233.5 (60.8)	233.6 (65.2)	226.6 (58.8)	258.4 (54.8)
<LLN, n (%)	3 (4.6%)	10 (3.1%)	1 (1.4%)	9 (6.3%)	0 (0%)
