# Supplementary material for: Integrated Assessment of Phase 2 Data on GalNAc3-Conjugated 2′-O-Methoxyethyl-Modified Antisense Oligonucleotides
Source: Nucleic Acid Ther. 2023 Feb 1;33(1):72–80. doi: 10.1089/nat.2022.0044 (PMC10623620; doi:10.1089/nat.2022.0044)
Supplement: Supplemental data [file Suppl_TableS4.pdf]

Supplemental Table 4. Baseline characteristics of patients in the weekly dose regime cohort

			Dose Category (mg/month)				
**Parameter**	**Placebo**	**Total ASO**	**>0 to <40**	**40 to <80**	**80 to <160**	**160 to <320**	**>=320**
**N**	65	259	23	71	80	35	50
**Age (yrs), Mean (SD)**	59.1 (9.6)	57.9 (10.7)	68.9 (6.8)	60.4 (11.1)	57.5 (8.4)	47.5 (9.6)	57.1 (9.2)
**Male, n (%)**	33 (50.8%)	166 (64.1%)	19 (82.6%)	49 (69.0%)	48 (60.0%)	23 (65.7%)	27 (54.0%)
**Race, n (%)**							
White	47 (72.3%)	190 (73.4%)	23 (100%)	70 (98.6%)	72 (90.0%)	0	25 (50.0%)
Asian	10 (15.4%)	57 (22.0%)	0	0	6 (7.5%)	35 (100%)	16 (32.0%)
Black	7 (10.8%)	10 (3.9%)	0	0	2 (2.5%)	0	8 (16.0%)
Other	1 (1.5%)	2 (0.8%)	0	1 (1.4%)	0	0	1 (2.0%)
**BMI (kg/m^2), Mean (SD)**	29.0 (5.0)	28.3 (5.0)	31.5 (3.6)	29.8 (4.5)	29.3 (5.2)	23.2 (2.6)	26.7 (4.2)
**Alanine Transaminase**							
Mean (SD), (U/L)	25.3 (15.4)	22.5 (10.1)	22.0 (7.5)	25.0 (11.4)	24.5 (10.4)	18.8 (7.7)	18.5 (8.2)
>ULN, N (%)	7 (10.8%)	13 (5%)	0 (0%)	7 (9.9%)	4 (5%)	1 (2.9%)	1 (2%)
**Serum Creatinine**							
Mean (SD), (mg/dL)	0.81 (0.18)	0.84 (0.19)	0.96 (0.13)	0.89 (0.19)	0.82 (0.19)	0.75 (0.15)	0.81 (0.20)
>ULN, N (%)	0 (0%)	0 (0%)	0 (0%)	0 (0%)	0 (0%)	0 (0%)	0 (0%)
**eGFR (CKD-EPI)**							
Mean (SD), mL/min/1.73 m^2	91.5 (14.9)	91.2 (16.4)	77.6 (12.1)	85.7 (16.0)	92.3 (16.2)	105.8 (9.4)	93.1 (14.4)
< 90 mL/min/1.73 m^2, n (%)	28 (43.1%)	109 (42.1%)	19 (82.6%)	41 (57%)	30 (37.5%)	1 (2.9%)	18 (36%)
< 60 mL/min/1.73 m^2, n (%)	1 (1.5%)	5 (1.9%)	1 (4.3%)	2 (2.8%)	2 (2.5%)	0 (0%)	0 (0%)
**Platelets**							
Mean (SD), k/uL	234.4 (63.5)	229.1 (57.8)	217.7 (49.7)	221.1 (53.8)	224.1 (55.1)	236.9 (60.5)	248.4 (65.8)
<LLN, n (%)	2 (3.1%)	13 (5%)	1 (4.3%)	3 (4.2%)	7 (8.7%)	0 (0%)	2 (4%)
