# Supplementary material for: Integrated Assessment of Phase 2 Data on GalNAc3-Conjugated 2′-O-Methoxyethyl-Modified Antisense Oligonucleotides
Source: Nucleic Acid Ther. 2023 Feb 1;33(1):72–80. doi: 10.1089/nat.2022.0044 (PMC10623620; doi:10.1089/nat.2022.0044)
Supplement: Supplemental data [file Suppl_TableS5.pdf]

Supplemental Table 5. Incidence of abnormalities in laboratory tests in the monthly dose regime cohort

				Dose Category (mg/month)		
	**Incidence of Events[*]**	**Placebo**	**Total ASO**	**>0 to <40**	**40 to <80**	**80 to <160**
**Liver**	**Alanine Transaminase (ALT)[†], n**	64	251	68	143	40
	> 3x ULN, or BL if > ULN	0	1 (0.4%)	1 (1.4%)	0	0
	> 5x ULN, or BL if > ULN	0	1 (0.4%)	1 (1.4%)	0	0
	**Aspartate Transaminase (AST)[†], n**	64	251	68	143	40
	> 3x ULN, or BL if > ULN	0	1 (0.4%)	1 (1.4%)	0	0
	> 5x ULN, or BL if > ULN	0	1 (0.4%)	1 (1.4%)	0	0
	**Albumin, n**	64	251	68	143	40
	< LLN, or BL if < LLN	0	0	0	0	0
	< 2.5 g/dL	0	0	0	0	0
	**Alkaline Phosphatase (ALP), n**	64	251	68	143	40
	> 3x ULN, or BL if > ULN	0	0	0	0	0
	**Total Bilirubin (TB), n**	64	251	68	143	40
	> 2x ULN, or BL if > ULN	0	0	0	0	0
	**Hy's Law[‡]**	64	251	68	143	40
	TB > 2x ULN and ALT > 3x ULN; or BL if > ULN	0	0	0	0	0
**Kidney**	**Serum Creatinine, n**	64	251	68	143	40
	≥ 0.3 mg/dL inc. from BL, or ≥ 1.5x BL	3 (4.6%)	6 (2.4%)	3 (4.3%)	1 (0.7%)	2 (5.0%)
	≥ 2x BL	0	0	0	0	0
	> 2.1 mg/dL	0	0	0	0	0
	**Blood Urea Nitrogen (BUN), n**	64	251	68	143	40
	≥ 2x ULN, or BL if > ULN	0	0	0	0	0
	**GFR CKD-EPI, n**	64	251	68	143	40
	< 60 mL/min per 1.73 m^2	5 (7.7%)	20 (7.9%)	8 (11.4%)	10 (7.0%)	2 (5.0%)
	< 30 mL/min per 1.73 m^2	0	0	0	0	0
	**Urine Protein, n**	64	251	68	143	40

	Incidence of Events*	Placebo	Total ASO	Dose Category (mg/month) >0 to <40	40 to <80	80 to <160
	≥ 2+ (100 mg/dL)	2 (3.1%)	8 (3.2%)	3 (4.3%)	4 (2.8%)	1 (2.5%)
	≥ 3+ (200 mg/dL)	0	0	0	0	0
Hematology	**Platelets, n**	64	251	68	143	40
	< 75 K/μL	0	0	0	0	0
	< 50 K/μL	0	0	0	0	0
	**Hemoglobin, n**	64	251	68	143	40
	M < 10.5 g/dL; F < 9.5 g/dL	0	2 (0.8%)	1 (1.4%)	1 (0.7%)	0
	**Hematocrit, n**	64	251	68	143	40
	< 0.85x BL	1 (1.5%)	10 (4.0%)	7 (10.0%)	3 (2.1%)	0
	< 30% (abs. value)	0	0	0	0	0
	**Lymphocytes, n**	64	250	68	143	39
	< 0.5 K/μL	0	0	0	0	0
	**Absolute Neutrophil Count (ANC), n**	64	250	68	143	39
	< 1.0 K/μL	0	0	0	0	0
Serum Electrolytes	**Potassium, n**	64	251	68	143	40
	< 3.0 mmol/L	0	0	0	0	0
	> 5.5 mmol/L	0	3 (1.2%)	0	2 (1.4%)	1 (2.5%)
	**Sodium, n**	64	251	68	143	40
	< 130 mmol/L	0	1 (0.4%)	1 (1.4%)	0	0
	> 150 mmol/L	0	0	0	0	0
	**Bicarbonate, n**	64	251	68	143	40
	< LLN, or BL if < LLN	1 (1.5%)	6 (2.4%)	2 (2.9%)	4 (2.8%)	0
	**Chloride, n**	64	251	68	143	40
	> ULN, or BL if > ULN	1 (1.5%)	0	0	0	0

* Results shown are confirmed events, defined as a consecutive abnormal lab value on next measurement after the initial observation and on a different day, unless specified otherwise. If there is no consecutive test to confirm, the initial observation is presumed confirmed.

† Elevated levels on two consecutive measurements at least 7 days apart with all values between the initial and subsequent test also above (or below) the specified threshold.

‡ ALT and Total Bilirubin must meet the criteria on the same day.
