# Supplementary material for: Integrated Assessment of Phase 2 Data on GalNAc3-Conjugated 2′-O-Methoxyethyl-Modified Antisense Oligonucleotides
Source: Nucleic Acid Ther. 2023 Feb 1;33(1):72–80. doi: 10.1089/nat.2022.0044 (PMC10623620; doi:10.1089/nat.2022.0044)
Supplement: Supplemental data [file Suppl_TableS9.pdf]

Supplemental Table 9. Liver lab test results over time by dose category for the monthly dose regime cohort. Tabulated summary of results for aspartate transaminase, total bilirubin, alkaline phosphatase, and albumin. Data shown represent at least 6 subjects and 2 GalNAc$_3$-conjugated ASOs. Pairwise comparison (vs placebo) is shown for the absolute change from baseline: *p < 0.05, †p < 0.01, ‡p < 0.001. Dose categories 160 to <320, and >=320 mg/month had no subjects from the monthly dose regimen cohort.

			Dose Category (mg/month)		
**Parameter**	**Visit**	**Placebo (N=65)**	**>0 to <40 (N=70)**	**40 to <80 (N=143)**	**80 to <160 (N=40)**
**Aspartate Transaminase, U/L**	**Screening**				
	Subjects, n	65	70	143	40
	ASO, n	4	2	3	2
	Mean (SD)	22.4 (9.0)	19.9 (6.2)	21.8 (8.2)	20.7 (7.4)
	**Baseline**				
	Subjects, n	65	70	143	40
	ASO, n	4	2	3	2
	Mean (SD)	23.2 (11.3)	19.5 (6.5)	21.2 (7.7)	18.6 (6.2)
	**Week 5**				
	Subjects, n	64	68	143	40
	ASO, n	4	2	3	2
	Mean (SD)	22.3 (9.5)	19.5 (6.4)	21.2 (7.0)	21.6 (9.1)
	Change from Baseline				
	Mean (SD)	-0.93 (5.44)	-0.04 (4.35)	0.08 (4.64)	3.02 (6.11)
	LSM	-0.79	-1.36	-0.59	3.11
	Diff in LSM		-0.58	0.19	3.89 ‡
	**Week 9**				
	Subjects, n	63	66	142	39
	ASO, n	4	2	3	2
	Mean (SD)	22.8 (11.0)	19.5 (6.2)	21.0 (6.5)	22.5 (10.6)
	Change from Baseline				
	Mean (SD)	-0.42 (3.87)	-0.20 (4.29)	-0.25 (5.75)	3.80 (7.24)
	LSM	-0.24	-1.05	-0.69	3.52
	Diff in LSM		-0.81	-0.44	3.76 †
	**Week 13**				
	Subjects, n	59	65	138	39
	ASO, n	4	2	3	2
	Mean (SD)	22.4 (9.3)	19.5 (6.5)	21.4 (7.2)	24.7 (12.0)
	Change from Baseline				
	Mean (SD)	-0.94 (5.34)	-0.14 (4.27)	0.10 (6.48)	5.93 (8.96)
	LSM	-0.70	-1.41	-0.60	5.69
	Diff in LSM		-0.71	0.11	6.39 ‡
	**Week 17**				
	Subjects, n	58	64	135	34

Parameter	Visit	Placebo (N=65)	Dose Category (mg/month) >0 to <40 (N=70)	40 to <80 (N=143)	80 to <160 (N=40)
	ASO, n	4	2	3	2
	Mean (SD)	21.9 (7.7)	19.6 (6.3)	21.7 (8.3)	24.1 (9.7)
	Change from Baseline				
	Mean (SD)	-1.63 (7.96)	0.03 (4.08)	0.31 (7.39)	5.53 (7.25)
	LSM	-1.06	-1.19	-0.18	4.56
	Diff in LSM		-0.13	0.87	5.62 ‡
	**Week 21**				
	Subjects, n	50	63	134	
	ASO, n	3	2	3	
	Mean (SD)	22.5 (9.6)	20.5 (10.1)	21.7 (8.8)	
	Change from Baseline				
	Mean (SD)	-1.49 (9.45)	0.83 (8.06)	0.55 (7.78)	
	LSM	-0.21	0.04	0.71	
	Diff in LSM		0.25	0.91	
	**Week 25**				
	Subjects, n	50	60	133	
	ASO, n	3	2	3	
	Mean (SD)	22.6 (7.0)	20.8 (9.4)	22.3 (8.5)	
	Change from Baseline				
	Mean (SD)	-1.75 (9.13)	0.90 (7.87)	1.02 (7.25)	
	LSM	-0.23	0.88	1.61	
	Diff in LSM		1.11	1.84	
	**Week 29**				
	Subjects, n	28	53	99	
	ASO, n	3	2	2	
	Mean (SD)	22.5 (6.5)	20.2 (7.8)	23.1 (9.6)	
	Change from Baseline				
	Mean (SD)	-1.71 (6.50)	0.77 (6.44)	0.74 (8.74)	
	LSM	-1.19	-0.73	0.65	
	Diff in LSM		0.46	1.84	
	**Week 33**				
	Subjects, n	24	39	77	
	ASO, n	2	2	2	
	Mean (SD)	23.1 (8.5)	19.7 (8.2)	22.1 (10.5)	
	Change from Baseline				
	Mean (SD)	-0.08 (7.05)	-0.02 (6.52)	0.40 (8.61)	
	LSM	0.66	-0.10	0.91	
	Diff in LSM		-0.76	0.25	
	**Week 37**				
	Subjects, n	19	33	66	

Parameter	Visit	Placebo (N=65)	Dose Category (mg/month) >0 to <40 (N=70)	40 to <80 (N=143)	80 to <160 (N=40)
	ASO, n	2	2	2	
	Mean (SD)	21.3 (6.1)	19.7 (8.0)	23.2 (13.6)	
	Change from Baseline				
	Mean (SD)	-1.89 (6.33)	-0.24 (6.53)	1.61 (11.21)	
	LSM	-1.22	-0.21	2.10	
	Diff in LSM		1.01	3.32	
	**Week 41**				
	Subjects, n	19	26	48	
	ASO, n	2	2	2	
	Mean (SD)	20.1 (4.8)	21.0 (10.2)	21.0 (8.8)	
	Change from Baseline				
	Mean (SD)	-2.13 (3.37)	1.37 (8.22)	0.43 (5.23)	
	LSM	-1.57	1.57	0.95	
	Diff in LSM		3.14	2.51	
	**Week 45**				
	Subjects, n	9	20	37	
	ASO, n	2	2	2	
	Mean (SD)	17.1 (3.8)	21.2 (11.5)	20.9 (7.1)	
	Change from Baseline				
	Mean (SD)	-4.11 (3.22)	1.95 (8.56)	-0.43 (5.48)	
	LSM	-3.84	1.78	0.29	
	Diff in LSM		5.62 *	4.14	
	**Week 49**				
	Subjects, n	6	14	24	
	ASO, n	2	2	2	
	Mean (SD)	18.2 (3.7)	20.7 (8.9)	21.7 (6.3)	
	Change from Baseline				
	Mean (SD)	-4.00 (3.41)	2.54 (6.36)	0.17 (6.11)	
	LSM	-3.77	1.38	0.61	
	Diff in LSM		5.15	4.37	
	**Week 53**				
	Subjects, n	6	14	15	
	ASO, n	2	2	2	
	Mean (SD)	18.2 (4.6)	20.9 (10.1)	22.9 (6.3)	
	Change from Baseline				
	Mean (SD)	-4.00 (2.19)	2.79 (5.06)	0.73 (5.06)	
	LSM	-4.37	2.31	0.72	
	Diff in LSM		6.67 †	5.08 *	
**Total Bilirubin, mg/dL**	**Screening**				

Parameter	Visit	Placebo	Dose Category (mg/month)		
		(N=65)	>0 to <40 (N=70)	40 to <80 (N=143)	80 to <160 (N=40)
	Subjects, n	65	70	143	40
	ASO, n	4	2	3	2
	Mean (SD)	0.58 (0.21)	0.55 (0.22)	0.63 (0.27)	0.56 (0.25)
	Baseline				
	Subjects, n	65	70	143	40
	ASO, n	4	2	3	2
	Mean (SD)	0.52 (0.19)	0.54 (0.25)	0.60 (0.24)	0.54 (0.22)
	**Week 5**				
	Subjects, n	64	68	143	40
	ASO, n	4	2	3	2
	Mean (SD)	0.54 (0.20)	0.54 (0.25)	0.60 (0.22)	0.50 (0.20)
	Change from Baseline				
	Mean (SD)	0.02 (0.09)	0.00 (0.11)	0.00 (0.15)	-0.04 (0.13)
	LSM	0.01	-0.02	-0.01	-0.02
	Diff in LSM		-0.03	-0.01	-0.03
	**Week 9**				
	Subjects, n	63	66	142	39
	ASO, n	4	2	3	2
	Mean (SD)	0.52 (0.18)	0.54 (0.25)	0.62 (0.26)	0.51 (0.22)
	Change from Baseline				
	Mean (SD)	0.00 (0.11)	-0.01 (0.15)	0.02 (0.17)	-0.03 (0.13)
	LSM	-0.02	-0.04	0.00	-0.01
	Diff in LSM		-0.02	0.02	0.01
	**Week 13**				
	Subjects, n	59	65	138	39
	ASO, n	4	2	3	2
	Mean (SD)	0.54 (0.19)	0.53 (0.25)	0.60 (0.23)	0.49 (0.21)
	Change from Baseline				
	Mean (SD)	0.01 (0.10)	-0.02 (0.13)	0.00 (0.17)	-0.05 (0.15)
	LSM	0.00	-0.04	0.00	-0.04
	Diff in LSM		-0.04	0.00	-0.05
	**Week 17**				
	Subjects, n	58	64	135	34
	ASO, n	4	2	3	2
	Mean (SD)	0.54 (0.21)	0.52 (0.23)	0.57 (0.22)	0.49 (0.26)
	Change from Baseline				
	Mean (SD)	0.02 (0.12)	-0.03 (0.12)	-0.02 (0.15)	-0.04 (0.15)
	LSM	0.00	-0.06	-0.03	-0.02
	Diff in LSM		-0.06 *	-0.03	-0.02
	**Week 21**				

Parameter	Visit	Placebo (N=65)	Dose Category (mg/month) >0 to <40 (N=70)	40 to <80 (N=143)	80 to <160 (N=40)
	Subjects, n	51	63	134	
	ASO, n	3	2	3	
	Mean (SD)	0.54 (0.23)	0.54 (0.27)	0.61 (0.24)	
	Change from Baseline				
	Mean (SD)	0.02 (0.16)	-0.01 (0.13)	0.01 (0.17)	
	LSM	0.00	-0.04	0.00	
	Diff in LSM		-0.04	0.00	
	**Week 25**				
	Subjects, n	50	60	133	
	ASO, n	3	2	3	
	Mean (SD)	0.58 (0.27)	0.55 (0.22)	0.60 (0.26)	
	Change from Baseline				
	Mean (SD)	0.05 (0.17)	0.00 (0.15)	0.00 (0.16)	
	LSM	0.04	-0.02	0.00	
	Diff in LSM		-0.06	-0.04	
	**Week 29**				
	Subjects, n	28	53	99	
	ASO, n	3	2	2	
	Mean (SD)	0.57 (0.20)	0.54 (0.27)	0.66 (0.32)	
	Change from Baseline				
	Mean (SD)	0.02 (0.13)	-0.01 (0.15)	0.04 (0.20)	
	LSM	0.00	-0.03	0.02	
	Diff in LSM		-0.03	0.02	
	**Week 33**				
	Subjects, n	24	39	79	
	ASO, n	2	2	2	
	Mean (SD)	0.61 (0.23)	0.57 (0.30)	0.58 (0.23)	
	Change from Baseline				
	Mean (SD)	0.04 (0.12)	-0.02 (0.16)	-0.03 (0.18)	
	LSM	0.02	-0.03	-0.04	
	Diff in LSM		-0.05	-0.06	
	**Week 37**				
	Subjects, n	19	33	66	
	ASO, n	2	2	2	
	Mean (SD)	0.60 (0.23)	0.56 (0.30)	0.59 (0.21)	
	Change from Baseline				
	Mean (SD)	0.01 (0.14)	-0.04 (0.16)	-0.02 (0.19)	
	LSM	0.00	-0.05	-0.03	
	Diff in LSM		-0.05	-0.03	
	**Week 41**				

Parameter	Visit	Placebo (N=65)	Dose Category (mg/month) >0 to <40 (N=70)	40 to <80 (N=143)	80 to <160 (N=40)
	Subjects, n	19	26	48	
	ASO, n	2	2	2	
	Mean (SD)	0.59 (0.21)	0.55 (0.29)	0.57 (0.19)	
	Change from Baseline				
	Mean (SD)	-0.01 (0.13)	-0.03 (0.14)	-0.04 (0.18)	
	LSM	-0.02	-0.04	-0.06	
	Diff in LSM		-0.02	-0.03	
	**Week 45**				
	Subjects, n	9	20	37	
	ASO, n	2	2	2	
	Mean (SD)	0.60 (0.21)	0.52 (0.15)	0.59 (0.22)	
	Change from Baseline				
	Mean (SD)	-0.03 (0.17)	0.00 (0.14)	-0.02 (0.21)	
	LSM	-0.01	-0.03	-0.02	
	Diff in LSM		-0.02	0.00	
	**Week 49**				
	Subjects, n	6	14	24	
	ASO, n	2	2	2	
	Mean (SD)	0.54 (0.09)	0.47 (0.11)	0.60 (0.21)	
	Change from Baseline				
	Mean (SD)	-0.06 (0.13)	-0.08 (0.18)	-0.02 (0.19)	
	LSM	-0.06	-0.11	-0.01	
	Diff in LSM		-0.05	0.05	
	**Week 53**				
	Subjects, n	6	14	15	
	ASO, n	2	2	2	
	Mean (SD)	0.58 (0.14)	0.53 (0.14)	0.58 (0.31)	
	Change from Baseline				
	Mean (SD)	-0.02 (0.15)	-0.02 (0.14)	0.00 (0.27)	
	LSM	0.01	-0.01	0.01	
	Diff in LSM		-0.02	0.00	
**Alkaline Phosphatase, U/L**	**Screening**				
	Subjects, n	65	70	143	40
	ASO, n	4	2	3	2
	Mean (SD)	68.5 (20.2)	64.5 (18.0)	64.6 (19.5)	76.7 (25.8)
	**Baseline**				
	Subjects, n	65	70	143	40
	ASO, n	4	2	3	2
	Mean (SD)	69.1 (21.4)	65.1 (19.3)	64.1 (18.3)	73.6 (24.6)

Parameter	Visit	Placebo (N=65)	Dose Category (mg/month) >0 to <40 (N=70)	40 to <80 (N=143)	80 to <160 (N=40)
	**Week 5**				
	Subjects, n	64	68	143	40
	ASO, n	4	2	3	2
	Mean (SD)	69.3 (21.3)	65.0 (19.1)	62.8 (17.9)	74.0 (24.4)
	Change from Baseline				
	Mean (SD)	0.02 (9.60)	-0.17 (6.93)	-1.35 (6.04)	0.35 (9.41)
	LSM	0.87	0.37	-0.65	1.05
	Diff in LSM		-0.50	-1.52	0.18
	**Week 9**				
	Subjects, n	63	66	142	39
	ASO, n	4	2	3	2
	Mean (SD)	68.7 (20.2)	64.5 (18.9)	61.9 (17.6)	73.6 (24.1)
	Change from Baseline				
	Mean (SD)	0.24 (10.16)	-0.77 (6.65)	-2.16 (7.01)	-0.67 (10.33)
	LSM	0.81	-0.65	-1.94	0.46
	Diff in LSM		-1.46	-2.75 *	-0.35
	**Week 13**				
	Subjects, n	59	65	138	39
	ASO, n	4	2	3	2
	Mean (SD)	67.4 (19.7)	63.6 (18.3)	62.3 (19.7)	72.7 (23.2)
	Change from Baseline				
	Mean (SD)	-1.95 (9.62)	-1.95 (8.34)	-2.03 (8.69)	-1.54 (12.06)
	LSM	-1.13	-1.42	-1.47	-0.73
	Diff in LSM		-0.29	-0.34	0.40
	**Week 17**				
	Subjects, n	58	64	135	34
	ASO, n	4	2	3	2
	Mean (SD)	66.2 (19.6)	64.0 (19.4)	61.8 (18.7)	73.3 (20.9)
	Change from Baseline				
	Mean (SD)	-2.81 (9.59)	-1.71 (9.05)	-2.49 (8.96)	-1.97 (10.38)
	LSM	-2.21	-1.38	-2.27	-0.95
	Diff in LSM		0.83	-0.06	1.26
	**Week 21**				
	Subjects, n	51	63	134	
	ASO, n	3	2	3	
	Mean (SD)	67.7 (19.5)	63.3 (19.0)	61.5 (19.0)	
	Change from Baseline				
	Mean (SD)	-3.45 (12.20)	-2.43 (8.29)	-2.91 (8.89)	
	LSM	-2.83	-2.19	-3.04	
	Diff in LSM		0.64	-0.21	

Parameter	Visit	Placebo (N=65)	Dose Category (mg/month) >0 to <40 (N=70)	40 to <80 (N=143)	80 to <160 (N=40)
	**Week 25**				
	Subjects, n	50	60	133	
	ASO, n	3	2	3	
	Mean (SD)	69.6 (20.7)	64.3 (19.9)	61.2 (19.0)	
	Change from Baseline				
	Mean (SD)	-1.39 (10.03)	-1.40 (10.05)	-3.01 (9.74)	
	LSM	-0.50	-0.77	-2.61	
	Diff in LSM		-0.26	-2.10	
	**Week 29**				
	Subjects, n	28	53	99	
	ASO, n	3	2	2	
	Mean (SD)	63.5 (17.5)	64.1 (22.1)	58.5 (17.4)	
	Change from Baseline				
	Mean (SD)	-1.79 (9.43)	-1.59 (12.07)	-4.19 (9.01)	
	LSM	-2.40	-2.24	-5.33	
	Diff in LSM		0.17	-2.93	
	**Week 33**				
	Subjects, n	24	39	78	
	ASO, n	2	2	2	
	Mean (SD)	60.8 (16.4)	61.5 (18.2)	61.0 (20.0)	
	Change from Baseline				
	Mean (SD)	-3.92 (6.54)	-3.07 (10.68)	-2.64 (9.86)	
	LSM	-3.66	-2.87	-2.45	
	Diff in LSM		0.79	1.21	
	**Week 37**				
	Subjects, n	19	33	66	
	ASO, n	2	2	2	
	Mean (SD)	62.2 (19.1)	64.5 (20.0)	61.3 (19.8)	
	Change from Baseline				
	Mean (SD)	-2.74 (7.33)	-1.56 (11.44)	-2.30 (11.72)	
	LSM	-1.99	-0.76	-1.54	
	Diff in LSM		1.22	0.45	
	**Week 41**				
	Subjects, n	19	26	48	
	ASO, n	2	2	2	
	Mean (SD)	62.0 (21.3)	61.5 (20.6)	60.8 (22.8)	
	Change from Baseline				
	Mean (SD)	-2.87 (7.28)	-2.98 (12.39)	-0.88 (17.28)	
	LSM	-2.55	-2.73	-0.92	
	Diff in LSM		-0.18	1.63	

Parameter	Visit	Placebo (N=65)	Dose Category (mg/month) >0 to <40 (N=70)	40 to <80 (N=143)	80 to <160 (N=40)
	**Week 45**				
	Subjects, n	9	20	37	
	ASO, n	2	2	2	
	Mean (SD)	56.3 (10.0)	61.1 (20.2)	60.3 (20.4)	
	Change from Baseline				
	Mean (SD)	-4.44 (10.49)	-1.60 (10.98)	-0.19 (12.02)	
	LSM	-4.51	-1.38	-0.30	
	Diff in LSM		3.14	4.21	
	**Week 49**				
	Subjects, n	6	14	24	
	ASO, n	2	2	2	
	Mean (SD)	59.8 (8.1)	63.2 (22.4)	63.1 (23.7)	
	Change from Baseline				
	Mean (SD)	-3.00 (9.19)	-0.36 (10.85)	-0.13 (11.22)	
	LSM	-2.88	-0.27	-0.15	
	Diff in LSM		2.62	2.73	
	**Week 53**				
	Subjects, n	6	14	15	
	ASO, n	2	2	2	
	Mean (SD)	60.0 (12.5)	63.1 (21.0)	67.3 (24.0)	
	Change from Baseline				
	Mean (SD)	-2.83 (9.00)	-0.43 (9.30)	0.00 (12.89)	
	LSM	-2.01	0.30	0.30	
	Diff in LSM		2.31	2.31	
**Albumin, g/dL**	**Screening**				
	Subjects, n	65	70	143	40
	ASO, n	4	2	3	2
	Mean (SD)	4.43 (0.28)	4.34 (0.26)	4.35 (0.27)	4.42 (0.23)
	**Baseline**				
	Subjects, n	65	70	143	40
	ASO, n	4	2	3	2
	Mean (SD)	4.34 (0.29)	4.28 (0.29)	4.28 (0.25)	4.33 (0.27)
	**Week 5**				
	Subjects, n	64	68	143	40
	ASO, n	4	2	3	2
	Mean (SD)	4.32 (0.25)	4.27 (0.24)	4.31 (0.24)	4.36 (0.26)
	Change from Baseline				
	Mean (SD)	0.00 (0.20)	-0.01 (0.17)	0.03 (0.17)	0.04 (0.18)
	LSM	0.02	0.00	0.04	0.04
	Diff in LSM		-0.01	0.03	0.03

Parameter	Visit	Placebo (N=65)	Dose Category (mg/month) >0 to <40 (N=70)	40 to <80 (N=143)	80 to <160 (N=40)
	**Week 9**				
	Subjects, n	63	66	142	39
	ASO, n	4	2	3	2
	Mean (SD)	4.32 (0.32)	4.29 (0.28)	4.31 (0.24)	4.32 (0.23)
	Change from Baseline				
	Mean (SD)	0.00 (0.24)	0.00 (0.21)	0.04 (0.18)	-0.01 (0.17)
	LSM	0.01	0.00	0.04	0.00
	Diff in LSM		-0.01	0.03	-0.01
	**Week 13**				
	Subjects, n	59	65	138	39
	ASO, n	4	2	3	2
	Mean (SD)	4.30 (0.25)	4.26 (0.26)	4.31 (0.23)	4.36 (0.26)
	Change from Baseline				
	Mean (SD)	-0.03 (0.19)	-0.02 (0.22)	0.03 (0.20)	0.03 (0.22)
	LSM	-0.01	-0.01	0.04	0.04
	Diff in LSM		0.00	0.05	0.05
	**Week 17**				
	Subjects, n	58	64	135	34
	ASO, n	4	2	3	2
	Mean (SD)	4.31 (0.22)	4.29 (0.26)	4.31 (0.24)	4.31 (0.22)
	Change from Baseline				
	Mean (SD)	-0.02 (0.20)	0.01 (0.20)	0.03 (0.21)	-0.01 (0.17)
	LSM	-0.01	-0.01	0.01	0.02
	Diff in LSM		0.00	0.03	0.03
	**Week 21**				
	Subjects, n	51	63	134	
	ASO, n	3	2	3	
	Mean (SD)	4.28 (0.29)	4.24 (0.26)	4.30 (0.25)	
	Change from Baseline				
	Mean (SD)	-0.03 (0.21)	-0.04 (0.21)	0.02 (0.19)	
	LSM	-0.02	-0.06	0.01	
	Diff in LSM		-0.04	0.03	
	**Week 25**				
	Subjects, n	50	60	133	
	ASO, n	3	2	3	
	Mean (SD)	4.36 (0.32)	4.27 (0.29)	4.32 (0.26)	
	Change from Baseline				
	Mean (SD)	0.05 (0.24)	0.00 (0.20)	0.03 (0.23)	
	LSM	0.06	0.01	0.04	
	Diff in LSM		-0.05	-0.02	

Parameter	Visit	Placebo (N=65)	Dose Category (mg/month) >0 to <40 (N=70)	40 to <80 (N=143)	80 to <160 (N=40)
	95% CI of Diff		-0.13, 0.03	-0.09, 0.05	
	P-value		0.2236	0.5932	
	**Week 29**				
	Subjects, n	28	53	99	
	ASO, n	3	2	2	
	Mean (SD)	4.27 (0.24)	4.27 (0.23)	4.31 (0.24)	
	Change from Baseline				
	Mean (SD)	-0.04 (0.17)	-0.02 (0.26)	0.05 (0.20)	
	LSM	-0.05	-0.05	0.01	
	Diff in LSM		0.01	0.06	
	**Week 33**				
	Subjects, n	24	39	79	
	ASO, n	2	2	2	
	Mean (SD)	4.25 (0.29)	4.18 (0.22)	4.25 (0.25)	
	Change from Baseline				
	Mean (SD)	-0.09 (0.15)	-0.10 (0.24)	-0.01 (0.19)	
	LSM	-0.06	-0.09	0.00	
	Diff in LSM		-0.03	0.06	
	**Week 37**				
	Subjects, n	19	33	66	
	ASO, n	2	2	2	
	Mean (SD)	4.31 (0.23)	4.28 (0.20)	4.27 (0.24)	
	Change from Baseline				
	Mean (SD)	-0.05 (0.23)	0.03 (0.23)	0.01 (0.20)	
	LSM	-0.02	0.01	0.00	
	Diff in LSM		0.03	0.01	
	**Week 41**				
	Subjects, n	19	26	48	
	ASO, n	2	2	2	
	Mean (SD)	4.27 (0.22)	4.21 (0.28)	4.24 (0.22)	
	Change from Baseline				
	Mean (SD)	-0.12 (0.17)	0.00 (0.19)	-0.01 (0.21)	
	LSM	-0.07	-0.02	-0.02	
	Diff in LSM		0.05	0.05	
	**Week 45**				
	Subjects, n	9	20	37	
	ASO, n	2	2	2	
	Mean (SD)	4.33 (0.25)	4.34 (0.29)	4.29 (0.22)	
	Change from Baseline				
	Mean (SD)	-0.07 (0.19)	0.12 (0.22)	0.03 (0.20)	

Parameter	Visit	Placebo (N=65)	Dose Category (mg/month) >0 to <40 (N=70)	40 to <80 (N=143)	80 to <160 (N=40)
	LSM	-0.02	0.11	0.03	
	Diff in LSM		0.12	0.05	
	95% CI of Diff		-0.03, 0.28	-0.09, 0.19	
	P-value		0.1127	0.4760	
	**Week 49**				
	Subjects, n	6	14	24	
	ASO, n	2	2	2	
	Mean (SD)	4.27 (0.31)	4.26 (0.26)	4.25 (0.19)	
	Change from Baseline				
	Mean (SD)	-0.10 (0.18)	0.01 (0.18)	0.02 (0.21)	
	LSM	-0.07	0.00	0.01	
	Diff in LSM		0.07	0.08	
	**Week 53**				
	Subjects, n	6	14	15	
	ASO, n	2	2	2	
	Mean (SD)	4.40 (0.36)	4.28 (0.30)	4.32 (0.25)	
	Change from Baseline				
	Mean (SD)	0.03 (0.22)	0.03 (0.25)	0.08 (0.23)	
	LSM	0.07	0.04	0.08	
	Diff in LSM		-0.03	0.01	

ASO denotes antisense oligonucleotide, SD denotes standard deviation. Least squares mean (LSM), difference in least squares means and p-values were estimated using an ANCOVA model with dose category and trial as fixed factors and baseline level as covariates.
