# Supplementary material for: Integrated Assessment of Phase 2 Data on GalNAc3-Conjugated 2′-O-Methoxyethyl-Modified Antisense Oligonucleotides
Source: Nucleic Acid Ther. 2023 Feb 1;33(1):72–80. doi: 10.1089/nat.2022.0044 (PMC10623620; doi:10.1089/nat.2022.0044)

Figure S2: Mean laboratory measurements over time for kidney tests by dose regime. The lower limit of normal (LLN) and upper limit of normal (ULN) displayed represent the median values. Screening was defined as average of all values measured prior to baseline. Each data point represents at least 6 subjects and 2 ASOs.

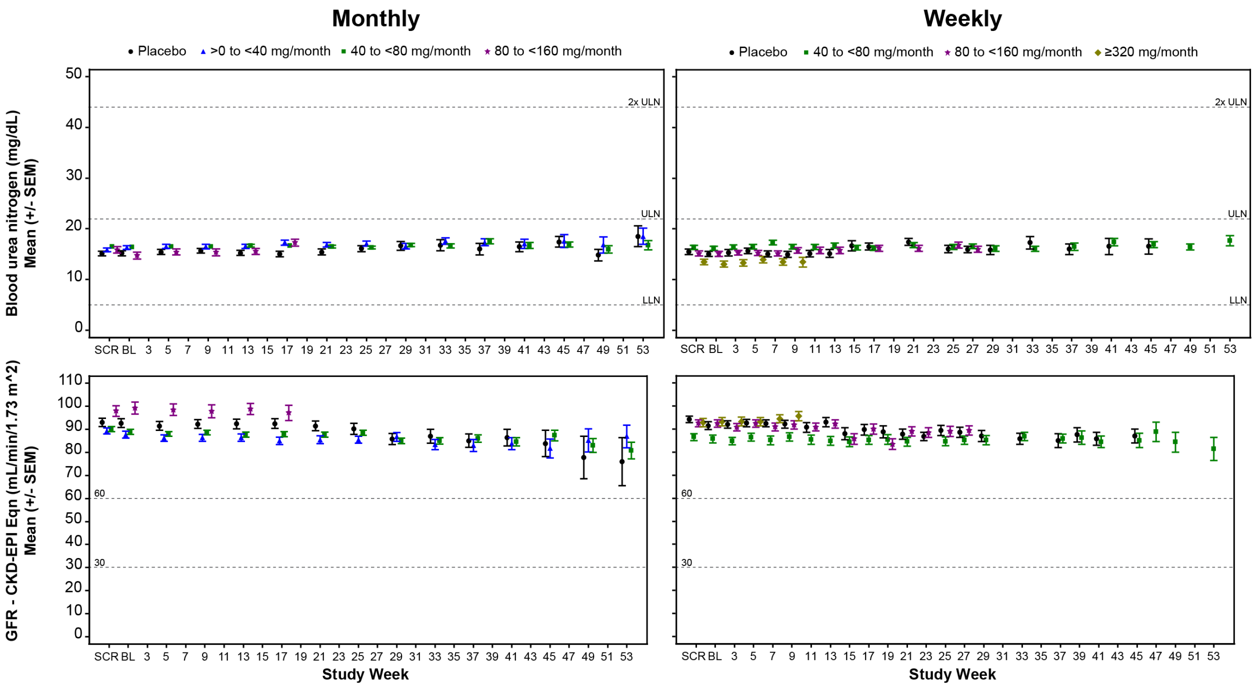

Supplement: Supplemental data [file Suppl_FigS2.pdf]
