# Supplementary material for: Integrated Assessment of Phase 2 Data on GalNAc3-Conjugated 2′-O-Methoxyethyl-Modified Antisense Oligonucleotides
Source: Nucleic Acid Ther. 2023 Feb 1;33(1):72–80. doi: 10.1089/nat.2022.0044 (PMC10623620; doi:10.1089/nat.2022.0044)
Supplement: Supplemental data [file Suppl_TableS16.pdf]

Supplemental Table 16: Serum electrolyte test results over time by dose category for the weekly dose regime cohort. Tabulated summary of results for potassium, sodium, bicarbonate, and chloride. Data shown represent at least 6 subjects and 2 GalNAc$_3$-conjugated ASOs. Pairwise comparison (vs placebo) is shown for the absolute change from baseline: *p < 0.05, †p < 0.01, ‡p < 0.001. Dose categories >0 to <40 (n=23) and 160 to <320 (n=35) mg/month represent a single ASO (data not shown).

			Dose Category (mg/month)		
**Parameter**	**Visit**	**Placebo (N=65)**	**40 to <80 (N=71)**	**80 to <160 (N=80)**	**>=320 (N=50)**
**Potassium,**	**Screening**				
**mEq/L**	Subjects, n	63	71	80	50
	ASO, n	6	2	3	3
	Mean (SD)	4.25 (0.30)	4.38 (0.36)	4.25 (0.34)	4.23 (0.30)
	**Baseline**				
	Subjects, n	65	71	80	50
	ASO, n	6	2	3	3
	Mean (SD)	4.23 (0.31)	4.28 (0.35)	4.24 (0.33)	4.18 (0.38)
	**Week 3**				
	Subjects, n	61	70	79	49
	ASO, n	6	2	3	3
	Mean (SD)	4.26 (0.34)	4.40 (0.39)	4.36 (0.37)	4.28 (0.32)
	Change from Baseline				
	Mean (SD)	0.03 (0.41)	0.13 (0.36)	0.12 (0.31)	0.11 (0.36)
	LSM	0.02	0.07	0.07	0.19
	Diff in LSM		0.04	0.05	0.16*
	**Week 5**				
	Subjects, n	60	69	76	48
	ASO, n	6	2	3	3
	Mean (SD)	4.26 (0.31)	4.36 (0.34)	4.34 (0.35)	4.24 (0.30)
	Change from Baseline				
	Mean (SD)	0.03 (0.29)	0.09 (0.29)	0.11 (0.31)	0.06 (0.39)
	LSM	0.03	0.09	0.07	0.09
	Diff in LSM		0.05	0.04	0.06
	**Week 7**				
	Subjects, n	58	67	74	46

Parameter	Visit	Placebo (N=65)	Dose Category (mg/month) 40 to <80 (N=71)	80 to <160 (N=80)	>=320 (N=50)
	ASO, n	6	2	3	3
	Mean (SD)	4.28 (0.32)	4.37 (0.43)	4.32 (0.35)	4.27 (0.31)
	Change from Baseline				
	Mean (SD)	0.05 (0.38)	0.10 (0.36)	0.10 (0.33)	0.10 (0.36)
	LSM	0.06	0.10	0.08	0.14
	Diff in LSM		0.05	0.02	0.08
	**Week 9**				
	Subjects, n	54	63	69	31
	ASO, n	5	2	3	2
	Mean (SD)	4.26 (0.29)	4.33 (0.40)	4.33 (0.35)	4.12 (0.30)
	Change from Baseline				
	Mean (SD)	0.05 (0.38)	0.09 (0.37)	0.08 (0.29)	-0.01 (0.29)
	LSM	0.04	0.04	0.05	0.06
	Diff in LSM		0.00	0.01	0.02
	**Week 11**				
	Subjects, n	47	62	67	
	ASO, n	4	2	3	
	Mean (SD)	4.33 (0.35)	4.34 (0.35)	4.35 (0.40)	
	Change from Baseline				
	Mean (SD)	0.13 (0.34)	0.08 (0.34)	0.10 (0.35)	
	LSM	0.13	0.11	0.11	
	Diff in LSM		-0.02	-0.01	
	**Week 13**				
	Subjects, n	45	62	66	
	ASO, n	4	2	3	
	Mean (SD)	4.29 (0.31)	4.32 (0.41)	4.27 (0.37)	
	Change from Baseline				
	Mean (SD)	0.09 (0.39)	0.06 (0.33)	0.02 (0.30)	
	LSM	0.08	0.06	0.05	
	Diff in LSM		-0.03	-0.03	
	**Week 15**				

Parameter	Visit	Placebo (N=65)	Dose Category (mg/month) 40 to <80 (N=71)	80 to <160 (N=80)	>=320 (N=50)
	Subjects, n	21	53		
	ASO, n	3	2		
	Mean (SD)	4.35 (0.28)	4.35 (0.38)		
	Change from Baseline				
	Mean (SD)	0.11 (0.31)	0.11 (0.37)		
	LSM	0.24	0.25		
	Diff in LSM		0.01		
	**Week 17**				
	Subjects, n	36	63	55	
	ASO, n	3	2	2	
	Mean (SD)	4.29 (0.29)	4.32 (0.34)	4.29 (0.34)	
	Change from Baseline				
	Mean (SD)	0.07 (0.27)	0.06 (0.28)	0.04 (0.30)	
	LSM	0.07	0.09	0.07	
	Diff in LSM		0.03	0.00	
	**Week 21**				
	Subjects, n	33	58	58	
	ASO, n	3	2	2	
	Mean (SD)	4.30 (0.32)	4.40 (0.41)	4.28 (0.35)	
	Change from Baseline				
	Mean (SD)	0.08 (0.29)	0.14 (0.33)	0.03 (0.31)	
	LSM	0.08	0.18	0.06	
	Diff in LSM		0.09	-0.03	
	**Week 25**				
	Subjects, n	35	57	54	
	ASO, n	3	2	2	
	Mean (SD)	4.35 (0.30)	4.37 (0.40)	4.33 (0.34)	
	Change from Baseline				
	Mean (SD)	0.13 (0.31)	0.09 (0.34)	0.09 (0.28)	
	LSM	0.11	0.08	0.07	
	Diff in LSM		-0.03	-0.05	

Parameter	Visit	Placebo (N=65)	Dose Category (mg/month) 40 to <80 (N=71)	80 to <160 (N=80)	>=320 (N=50)
	**Week 27**				
	Subjects, n	34	58	53	
	ASO, n	3	2	2	
	Mean (SD)	4.29 (0.35)	4.23 (0.31)	4.32 (0.38)	
	Change from Baseline				
	Mean (SD)	0.07 (0.39)	-0.05 (0.31)	0.05 (0.29)	
	LSM	0.06	-0.01	0.08	
	Diff in LSM		-0.07	0.02	
	**Week 29**				
	Subjects, n	25	51		
	ASO, n	3	2		
	Mean (SD)	4.28 (0.33)	4.32 (0.33)		
	Change from Baseline				
	Mean (SD)	0.11 (0.43)	0.08 (0.28)		
	LSM	0.01	0.01		
	Diff in LSM		0.00		
	**Week 33**				
	Subjects, n	18	44		
	ASO, n	2	2		
	Mean (SD)	4.34 (0.25)	4.35 (0.38)		
	Change from Baseline				
	Mean (SD)	0.16 (0.28)	0.13 (0.31)		
	LSM	0.16	0.15		
	Diff in LSM		-0.01		
	**Week 37**				
	Subjects, n	15	35		
	ASO, n	2	2		
	Mean (SD)	4.33 (0.41)	4.33 (0.37)		
	Change from Baseline				
	Mean (SD)	0.21 (0.29)	0.08 (0.34)		
	LSM	0.18	0.11		

Parameter	Visit	Placebo (N=65)	Dose Category (mg/month)		
			40 to <80 (N=71)	80 to <160 (N=80)	>=320 (N=50)
	Diff in LSM		-0.07		
	**Week 41**				
	Subjects, n	11	29		
	ASO, n	2	2		
	Mean (SD)	4.25 (0.40)	4.29 (0.33)		
	Change from Baseline				
	Mean (SD)	0.19 (0.28)	0.04 (0.32)		
	LSM	0.17	0.08		
	Diff in LSM		-0.08		
	**Week 45**				
	Subjects, n	9	22		
	ASO, n	2	2		
	Mean (SD)	4.26 (0.26)	4.23 (0.33)		
	Change from Baseline				
	Mean (SD)	0.21 (0.24)	0.01 (0.29)		
	LSM	0.19	0.04		
	Diff in LSM		-0.14		
	**Week 49**				
	Subjects, n		17		
	ASO, n		2		
	Mean (SD)		4.32 (0.38)		
	Change from Baseline				
	Mean (SD)		0.18 (0.41)		
	LSM	NA	0.18		
	Diff in LSM		NA		
	**Week 53**				
	Subjects, n		13		
	ASO, n		2		
	Mean (SD)		4.18 (0.32)		
	Change from Baseline				
	Mean (SD)		0.05 (0.26)		

Parameter	Visit	Placebo (N=65)	Dose Category (mg/month) 40 to <80 (N=71)	80 to <160 (N=80)	>=320 (N=50)
	LSM		0.04		
	Diff in LSM		NA		
**Sodium, mEq/L**	**Screening**				
	Subjects, n	64	71	80	50
	ASO, n	6	2	3	3
	Mean (SD)	138.7 (2.8)	139.3 (2.4)	139.0 (2.0)	139.0 (3.1)
	**Baseline**				
	Subjects, n	65	71	80	50
	ASO, n	6	2	3	3
	Mean (SD)	138.6 (2.7)	138.9 (2.5)	138.5 (2.3)	139.2 (3.4)
	**Week 3**				
	Subjects, n	61	70	80	49
	ASO, n	6	2	3	3
	Mean (SD)	138.8 (2.3)	139.0 (2.4)	138.4 (2.6)	139.1 (2.7)
	Change from Baseline				
	Mean (SD)	0.08 (2.04)	0.16 (1.96)	-0.03 (2.22)	-0.09 (1.82)
	LSM	-0.06	0.30	-0.01	-0.37
	Diff in LSM		0.36	0.05	-0.31
	**Week 5**				
	Subjects, n	60	69	76	48
	ASO, n	6	2	3	3
	Mean (SD)	139.0 (2.8)	138.7 (2.5)	139.0 (2.5)	138.7 (3.0)
	Change from Baseline				
	Mean (SD)	0.28 (2.33)	-0.12 (1.90)	0.52 (2.26)	-0.65 (1.84)
	LSM	0.06	-0.14	0.18	-0.85
	Diff in LSM		-0.20	0.12	-0.91*
	**Week 7**				
	Subjects, n	58	68	74	46
	ASO, n	6	2	3	3
	Mean (SD)	139.3 (2.5)	138.7 (2.6)	138.8 (2.6)	138.7 (3.6)
	Change from Baseline				

Parameter	Visit	Placebo (N=65)	Dose Category (mg/month) 40 to <80 (N=71)	80 to <160 (N=80)	>=320 (N=50)
	Mean (SD)	0.61 (2.10)	-0.13 (2.52)	0.31 (2.00)	-0.67 (2.06)
	LSM	0.35	-0.17	0.31	-0.68
	Diff in LSM		-0.52	-0.05	-1.04*
	**Week 9**				
	Subjects, n	54	63	69	31
	ASO, n	5	2	3	2
	Mean (SD)	139.3 (2.3)	138.7 (2.5)	139.0 (2.3)	138.9 (3.6)
	Change from Baseline				
	Mean (SD)	0.68 (2.17)	-0.11 (2.27)	0.46 (1.88)	-0.48 (2.15)
	LSM	0.37	-0.49	0.26	-0.38
	Diff in LSM		-0.86*	-0.11	-0.75
	**Week 11**				
	Subjects, n	47	62	68	
	ASO, n	4	2	3	
	Mean (SD)	139.6 (2.2)	138.9 (2.6)	138.6 (2.1)	
	Change from Baseline				
	Mean (SD)	0.98 (1.72)	0.13 (2.45)	-0.01 (2.07)	
	LSM	0.70	-0.07	-0.04	
	Diff in LSM		-0.76*	-0.73*	
	**Week 13**				
	Subjects, n	45	62	66	
	ASO, n	4	2	3	
	Mean (SD)	139.0 (2.5)	138.6 (2.5)	138.8 (2.9)	
	Change from Baseline				
	Mean (SD)	0.44 (1.80)	-0.11 (2.10)	0.33 (2.27)	
	LSM	0.21	-0.24	0.32	
	Diff in LSM		-0.44	0.11	
	**Week 15**				
	Subjects, n	21	53		
	ASO, n	3	2		
	Mean (SD)	138.5 (1.9)	139.0 (2.6)		

Parameter	Visit	Placebo (N=65)	Dose Category (mg/month) 40 to <80 (N=71)	80 to <160 (N=80)	>=320 (N=50)
	Change from Baseline				
	Mean (SD)	0.60 (2.08)	0.08 (2.16)		
	LSM	0.40	0.22		
	Diff in LSM		-0.18		
	**Week 17**				
	Subjects, n	36	63	55	
	ASO, n	3	2	2	
	Mean (SD)	138.1 (2.3)	138.9 (2.5)	138.1 (2.7)	
	Change from Baseline				
	Mean (SD)	0.33 (1.79)	0.12 (2.62)	-0.16 (2.48)	
	LSM	-0.03	-0.05	-0.17	
	Diff in LSM		-0.02	-0.13	
	**Week 21**				
	Subjects, n	33	58	58	
	ASO, n	3	2	2	
	Mean (SD)	138.6 (2.2)	139.0 (2.5)	138.5 (2.2)	
	Change from Baseline				
	Mean (SD)	0.73 (2.27)	0.27 (2.42)	0.06 (1.96)	
	LSM	0.36	0.24	-0.13	
	Diff in LSM		-0.11	-0.48	
	**Week 25**				
	Subjects, n	35	57	55	
	ASO, n	3	2	2	
	Mean (SD)	138.3 (2.2)	138.6 (2.4)	138.6 (2.5)	
	Change from Baseline				
	Mean (SD)	0.60 (2.24)	-0.11 (2.30)	0.34 (1.90)	
	LSM	0.23	-0.34	0.31	
	Diff in LSM		-0.57	0.08	
	**Week 27**				
	Subjects, n	34	58	53	
	ASO, n	3	2	2	

Parameter	Visit	Placebo (N=65)	Dose Category (mg/month)		
			40 to <80 (N=71)	80 to <160 (N=80)	>=320 (N=50)
	Mean (SD)	138.9 (2.2)	138.9 (2.2)	138.7 (2.4)	
	Change from Baseline				
	Mean (SD)	1.18 (1.96)	0.04 (2.35)	0.57 (2.31)	
	LSM	0.91	0.39	0.63	
	Diff in LSM		-0.53	-0.29	
	**Week 29**				
	Subjects, n	25	51		
	ASO, n	3	2		
	Mean (SD)	139.2 (2.1)	139.0 (2.4)		
	Change from Baseline				
	Mean (SD)	1.54 (1.84)	0.17 (2.12)		
	LSM	0.44	-0.61		
	Diff in LSM		-1.05*		
	**Week 33**				
	Subjects, n	18	44		
	ASO, n	2	2		
	Mean (SD)	138.7 (2.3)	138.6 (2.5)		
	Change from Baseline				
	Mean (SD)	1.33 (2.03)	-0.25 (2.08)		
	LSM	1.02	0.00		
	Diff in LSM		-1.02		
	**Week 37**				
	Subjects, n	15	35		
	ASO, n	2	2		
	Mean (SD)	138.5 (1.6)	138.9 (2.3)		
	Change from Baseline				
	Mean (SD)	1.07 (2.02)	-0.09 (2.37)		
	LSM	0.74	0.15		
	Diff in LSM		-0.59		
	**Week 41**				
	Subjects, n	11	29		

Parameter	Visit	Placebo (N=65)	Dose Category (mg/month) 40 to <80 (N=71)	80 to <160 (N=80)	>=320 (N=50)
	ASO, n	2	2		
	Mean (SD)	138.5 (1.6)	138.7 (2.3)		
	Change from Baseline				
	Mean (SD)	1.27 (1.79)	-0.34 (2.72)		
	LSM	0.61	-0.17		
	Diff in LSM		-0.78		
	**Week 45**				
	Subjects, n	9	22		
	ASO, n	2	2		
	Mean (SD)	139.0 (1.7)	139.1 (3.2)		
	Change from Baseline				
	Mean (SD)	1.22 (1.99)	0.11 (3.07)		
	LSM	1.03	0.08		
	Diff in LSM		-0.95		
	**Week 49**				
	Subjects, n		17		
	ASO, n		2		
	Mean (SD)		139.1 (2.1)		
	Change from Baseline				
	Mean (SD)		0.12 (2.50)		
	LSM		0.01		
	Diff in LSM		NA		
	**Week 53**				
	Subjects, n		13		
	ASO, n		2		
	Mean (SD)		138.5 (2.0)		
	Change from Baseline				
	Mean (SD)		-0.46 (3.55)		
	LSM		-0.67		
	Diff in LSM		NA		
**Bicarbonate,**	**Screening**				

Parameter	Visit	Placebo (N=65)	Dose Category (mg/month) 40 to <80 (N=71)	80 to <160 (N=80)	>=320 (N=50)
**mEq/L**	Subjects, n	54	71	74	35
	ASO, n	5	2	2	2
	Mean (SD)	25.7 (3.0)	25.1 (2.2)	25.8 (2.2)	27.8 (1.8)
	**Baseline**				
	Subjects, n	55	71	74	35
	ASO, n	5	2	2	2
	Mean (SD)	26.5 (2.1)	25.7 (2.4)	26.2 (2.5)	27.0 (2.0)
	**Week 3**				
	Subjects, n	51	70	74	34
	ASO, n	5	2	2	2
	Mean (SD)	27.1 (2.8)	25.9 (2.2)	26.7 (2.0)	28.5 (2.1)
	Change from Baseline				
	Mean (SD)	0.48 (2.46)	0.26 (2.41)	0.45 (2.41)	1.44 (2.29)
	LSM	0.78	0.61	0.90	1.09
	Diff in LSM		-0.17	0.11	0.30
	**Week 5**				
	Subjects, n	50	69	70	33
	ASO, n	5	2	2	2
	Mean (SD)	26.9 (2.8)	26.2 (2.3)	26.8 (1.9)	28.3 (1.9)
	Change from Baseline				
	Mean (SD)	0.23 (2.27)	0.59 (2.12)	0.54 (2.36)	1.14 (2.32)
	LSM	0.55	0.90	1.07	0.38
	Diff in LSM		0.36	0.52	-0.17
	**Week 7**				
	Subjects, n	48	68	68	31
	ASO, n	5	2	2	2
	Mean (SD)	26.4 (3.5)	26.1 (2.2)	26.6 (2.3)	28.4 (2.1)
	Change from Baseline				
	Mean (SD)	-0.18 (2.65)	0.38 (2.44)	0.38 (2.81)	1.01 (2.08)
	LSM	0.21	1.13	1.12	0.12
	Diff in LSM		0.91	0.91	-0.09

Parameter	Visit	Placebo (N=65)	Dose Category (mg/month) 40 to <80 (N=71)	80 to <160 (N=80)	>=320 (N=50)
	**Week 9**				
	Subjects, n	44	63	63	
	ASO, n	4	2	2	
	Mean (SD)	26.3 (3.3)	25.8 (2.3)	26.3 (2.2)	
	Change from Baseline				
	Mean (SD)	-0.23 (2.62)	0.05 (2.16)	-0.03 (2.36)	
	LSM	0.21	1.07	0.85	
	Diff in LSM		0.86	0.64	
	**Week 11**				
	Subjects, n	37	62	62	
	ASO, n	3	2	2	
	Mean (SD)	26.4 (2.5)	26.2 (2.6)	26.5 (2.3)	
	Change from Baseline				
	Mean (SD)	0.26 (2.52)	0.48 (2.59)	0.21 (2.62)	
	LSM	0.27	0.40	0.03	
	Diff in LSM		0.13	-0.24	
	**Week 13**				
	Subjects, n	35	62	60	
	ASO, n	3	2	2	
	Mean (SD)	25.6 (2.4)	26.2 (2.4)	26.6 (2.6)	
	Change from Baseline				
	Mean (SD)	-0.53 (2.05)	0.43 (2.20)	0.33 (2.30)	
	LSM	-0.57	0.17	0.17	
	Diff in LSM		0.75	0.74	
	**Week 15**				
	Subjects, n	21	53		
	ASO, n	3	2		
	Mean (SD)	26.9 (2.7)	26.2 (2.5)		
	Change from Baseline				
	Mean (SD)	0.43 (2.84)	0.37 (2.60)		
	LSM	1.80	1.65		

Parameter	Visit	Placebo (N=65)	Dose Category (mg/month) 40 to <80 (N=71)	80 to <160 (N=80)	>=320 (N=50)
	Diff in LSM		-0.16		
	**Week 17**				
	Subjects, n	36	63	55	
	ASO, n	3	2	2	
	Mean (SD)	25.8 (1.8)	26.3 (2.4)	26.5 (2.1)	
	Change from Baseline				
	Mean (SD)	-0.21 (1.88)	0.56 (2.29)	0.05 (2.08)	
	LSM	-0.34	0.19	-0.05	
	Diff in LSM		0.52	0.29	
	**Week 21**				
	Subjects, n	33	58	58	
	ASO, n	3	2	2	
	Mean (SD)	26.2 (1.6)	26.5 (2.4)	26.3 (2.4)	
	Change from Baseline				
	Mean (SD)	-0.06 (1.90)	0.69 (2.25)	-0.11 (2.30)	
	LSM	-0.07	0.64	-0.20	
	Diff in LSM		0.71	-0.13	
	**Week 25**				
	Subjects, n	35	57	55	
	ASO, n	3	2	2	
	Mean (SD)	26.5 (2.3)	26.2 (2.2)	26.7 (2.3)	
	Change from Baseline				
	Mean (SD)	0.40 (2.25)	0.37 (2.60)	0.36 (2.23)	
	LSM	0.34	0.17	0.36	
	Diff in LSM		-0.17	0.02	
	**Week 27**				
	Subjects, n	34	58	53	
	ASO, n	3	2	2	
	Mean (SD)	25.8 (2.4)	25.7 (2.6)	26.6 (2.5)	
	Change from Baseline				
	Mean (SD)	-0.29 (2.26)	-0.01 (2.26)	0.30 (2.06)	

Parameter	Visit	Placebo (N=65)	Dose Category (mg/month) 40 to <80 (N=71)	80 to <160 (N=80)	>=320 (N=50)
	LSM	-0.34	-0.20	0.22	
	Diff in LSM		0.14	0.57	
	**Week 29**				
	Subjects, n	25	51		
	ASO, n	3	2		
	Mean (SD)	25.9 (2.6)	26.0 (2.6)		
	Change from Baseline				
	Mean (SD)	-0.34 (2.40)	0.26 (2.09)		
	LSM	0.14	0.52		
	Diff in LSM		0.37		
	**Week 33**				
	Subjects, n	18	44		
	ASO, n	2	2		
	Mean (SD)	25.9 (2.2)	26.1 (2.0)		
	Change from Baseline				
	Mean (SD)	-0.44 (2.04)	0.09 (2.27)		
	LSM	-0.48	-0.06		
	Diff in LSM		0.42		
	**Week 37**				
	Subjects, n	15	35		
	ASO, n	2	2		
	Mean (SD)	25.5 (2.5)	26.2 (2.4)		
	Change from Baseline				
	Mean (SD)	-0.80 (2.46)	0.57 (2.13)		
	LSM	-0.80	0.37		
	Diff in LSM		1.17		
	**Week 41**				
	Subjects, n	11	29		
	ASO, n	2	2		
	Mean (SD)	26.4 (2.7)	25.9 (2.2)		
	Change from Baseline				

Parameter	Visit	Placebo (N=65)	Dose Category (mg/month) 40 to <80 (N=71)	80 to <160 (N=80)	>=320 (N=50)
	Mean (SD)	0.45 (2.02)	0.21 (2.24)		
	LSM	0.45	0.14		
	Diff in LSM		-0.31		
	**Week 45**				
	Subjects, n	9	22		
	ASO, n	2	2		
	Mean (SD)	25.4 (3.5)	25.6 (2.0)		
	Change from Baseline				
	Mean (SD)	-0.78 (2.05)	0.32 (1.89)		
	LSM	-0.80	0.04		
	Diff in LSM		0.84		
	**Week 49**				
	Subjects, n		17		
	ASO, n		2		
	Mean (SD)		25.9 (1.9)		
	Change from Baseline				
	Mean (SD)		0.53 (2.21)		
	LSM		0.21		
	Diff in LSM		NA		
	**Week 53**				
	Subjects, n		13		
	ASO, n		2		
	Mean (SD)		25.2 (2.1)		
	Change from Baseline				
	Mean (SD)		0.00 (2.38)		
	LSM		0.30		
	Diff in LSM		NA		
**Chloride, mEq/L**	**Screening**				
	Subjects, n	54	71	74	35
	ASO, n	5	2	2	2
	Mean (SD)	102.6 (2.8)	104.1 (2.9)	103.3 (2.7)	101.4 (2.9)

Parameter	Visit	Placebo (N=65)	Dose Category (mg/month) 40 to <80 (N=71)	80 to <160 (N=80)	>=320 (N=50)
	**Baseline**				
	Subjects, n	55	71	74	35
	ASO, n	5	2	2	2
	Mean (SD)	102.4 (2.8)	103.8 (2.8)	102.9 (2.8)	102.1 (4.4)
	**Week 3**				
	Subjects, n	51	70	74	34
	ASO, n	5	2	2	2
	Mean (SD)	102.6 (2.5)	104.1 (2.5)	103.1 (2.7)	102.0 (3.5)
	Change from Baseline				
	Mean (SD)	0.22 (2.24)	0.23 (2.21)	0.22 (2.01)	0.04 (2.20)
	LSM	0.06	0.54	0.27	-0.65
	Diff in LSM		0.47	0.21	-0.72
	**Week 5**				
	Subjects, n	50	69	70	33
	ASO, n	5	2	2	2
	Mean (SD)	102.9 (2.8)	104.0 (2.6)	103.4 (2.3)	101.8 (3.3)
	Change from Baseline				
	Mean (SD)	0.63 (2.42)	0.14 (2.07)	0.58 (2.30)	-0.30 (2.47)
	LSM	0.28	0.15	0.21	-0.56
	Diff in LSM		-0.14	-0.08	-0.84
	**Week 7**				
	Subjects, n	48	68	68	31
	ASO, n	5	2	2	2
	Mean (SD)	103.2 (2.6)	103.9 (2.9)	103.2 (2.4)	101.3 (4.0)
	Change from Baseline				
	Mean (SD)	0.79 (2.16)	0.07 (2.16)	0.29 (2.35)	-0.81 (2.23)
	LSM	0.52	0.08	0.17	-0.90
	Diff in LSM		-0.44	-0.35	-1.42*
	**Week 9**				
	Subjects, n	44	63	63	
	ASO, n	4	2	2	

Parameter	Visit	Placebo (N=65)	Dose Category (mg/month) 40 to <80 (N=71)	80 to <160 (N=80)	>=320 (N=50)
	Mean (SD)	103.2 (2.6)	103.7 (2.8)	103.2 (2.6)	
	Change from Baseline				
	Mean (SD)	0.98 (2.30)	-0.13 (1.95)	0.31 (2.11)	
	LSM	0.65	-0.29	0.26	
	Diff in LSM		-0.94*	-0.39	
	**Week 11**				
	Subjects, n	37	62	62	
	ASO, n	3	2	2	
	Mean (SD)	103.4 (2.7)	104.0 (2.6)	103.1 (2.7)	
	Change from Baseline				
	Mean (SD)	1.15 (1.80)	0.23 (2.29)	0.23 (2.28)	
	LSM	0.81	0.24	0.19	
	Diff in LSM		-0.57	-0.62	
	**Week 13**				
	Subjects, n	35	62	60	
	ASO, n	3	2	2	
	Mean (SD)	103.2 (2.9)	103.7 (3.2)	103.1 (3.9)	
	Change from Baseline				
	Mean (SD)	0.83 (2.24)	-0.09 (2.89)	0.16 (2.74)	
	LSM	0.64	-0.14	0.15	
	Diff in LSM		-0.79	-0.50	
	**Week 15**				
	Subjects, n	21	53		
	ASO, n	3	2		
	Mean (SD)	103.6 (2.9)	104.0 (2.8)		
	Change from Baseline				
	Mean (SD)	0.60 (2.41)	0.14 (2.14)		
	LSM	0.25	-0.04		
	Diff in LSM		-0.29		
	**Week 17**				
	Subjects, n	36	63	55	

Parameter	Visit	Placebo (N=65)	Dose Category (mg/month) 40 to <80 (N=71)	80 to <160 (N=80)	>=320 (N=50)
	ASO, n	3	2	2	
	Mean (SD)	102.9 (2.5)	103.9 (2.6)	102.6 (3.6)	
	Change from Baseline				
	Mean (SD)	0.54 (2.12)	0.09 (2.51)	-0.17 (2.54)	
	LSM	0.28	0.06	-0.32	
	Diff in LSM		-0.22	-0.60	
	**Week 21**				
	Subjects, n	33	58	58	
	ASO, n	3	2	2	
	Mean (SD)	103.0 (2.4)	103.6 (2.9)	102.8 (2.9)	
	Change from Baseline				
	Mean (SD)	0.68 (2.21)	-0.05 (2.67)	-0.08 (2.03)	
	LSM	0.33	-0.13	-0.36	
	Diff in LSM		-0.46	-0.69	
	**Week 25**				
	Subjects, n	35	57	55	
	ASO, n	3	2	2	
	Mean (SD)	102.8 (3.2)	103.9 (2.5)	102.8 (2.7)	
	Change from Baseline				
	Mean (SD)	0.54 (2.27)	0.12 (2.49)	0.07 (2.18)	
	LSM	0.20	-0.05	-0.02	
	Diff in LSM		-0.26	-0.23	
	**Week 27**				
	Subjects, n	34	58	53	
	ASO, n	3	2	2	
	Mean (SD)	103.4 (2.6)	104.1 (2.4)	102.8 (3.2)	
	Change from Baseline				
	Mean (SD)	1.06 (2.13)	0.12 (2.32)	-0.01 (2.02)	
	LSM	0.75	0.09	-0.36	
	Diff in LSM		-0.67	-1.11*	
	**Week 29**				

Parameter	Visit	Placebo (N=65)	Dose Category (mg/month) 40 to <80 (N=71)	80 to <160 (N=80)	>=320 (N=50)
	Subjects, n	25	51		
	ASO, n	3	2		
	Mean (SD)	104.1 (2.5)	104.1 (2.7)		
	Change from Baseline				
	Mean (SD)	1.42 (2.07)	0.32 (1.91)		
	LSM	1.41	0.59		
	Diff in LSM		-0.82		
	**Week 33**				
	Subjects, n	18	44		
	ASO, n	2	2		
	Mean (SD)	103.7 (2.1)	103.9 (3.1)		
	Change from Baseline				
	Mean (SD)	0.72 (2.02)	0.09 (2.14)		
	LSM	0.64	0.17		
	Diff in LSM		-0.47		
	**Week 37**				
	Subjects, n	15	35		
	ASO, n	2	2		
	Mean (SD)	102.9 (1.7)	103.4 (3.2)		
	Change from Baseline				
	Mean (SD)	0.33 (2.09)	-0.60 (1.96)		
	LSM	-0.03	-0.60		
	Diff in LSM		-0.57		
	**Week 41**				
	Subjects, n	11	29		
	ASO, n	2	2		
	Mean (SD)	103.8 (2.3)	103.6 (3.0)		
	Change from Baseline				
	Mean (SD)	1.09 (2.17)	-0.21 (2.44)		
	LSM	0.81	-0.18		
	Diff in LSM		-0.98		

Parameter	Visit	Placebo (N=65)	Dose Category (mg/month)		
			40 to <80 (N=71)	80 to <160 (N=80)	>=320 (N=50)
	**Week 45**				
	Subjects, n	9	22		
	ASO, n	2	2		
	Mean (SD)	103.8 (2.8)	103.8 (3.8)		
	Change from Baseline				
	Mean (SD)	0.67 (2.00)	-0.03 (2.77)		
	LSM	0.64	0.05		
	Diff in LSM		-0.59		
	**Week 49**				
	Subjects, n		17		
	ASO, n		2		
	Mean (SD)		103.9 (3.1)		
	Change from Baseline				
	Mean (SD)		0.06 (2.16)		
	LSM		0.18		
	Diff in LSM		NA		
	**Week 53**				
	Subjects, n		13		
	ASO, n		2		
	Mean (SD)		103.5 (3.1)		
	Change from Baseline				
	Mean (SD)		-0.31 (2.63)		
	LSM		-0.34		
	Diff in LSM		NA		

ASO denotes antisense oligonucleotide, SD denotes standard deviation. Least squares mean (LSM), difference in least squares means and p-values were estimated using an ANCOVA model with dose category and trial as fixed factors and baseline level as covariates.
