# Supplementary material for: Integrated Assessment of Phase 2 Data on GalNAc3-Conjugated 2′-O-Methoxyethyl-Modified Antisense Oligonucleotides
Source: Nucleic Acid Ther. 2023 Feb 1;33(1):72–80. doi: 10.1089/nat.2022.0044 (PMC10623620; doi:10.1089/nat.2022.0044)
Supplement: Supplemental data [file Suppl_TableS17.pdf]

Supplemental Table 17. Tolerability profile of $GalNAc_3$-conjugated antisense oligonucleotides in total study population

			Dose (mg/month)				
	**Placebo**	**Total ASO**	**>0 to <40**	**40 to <80**	**80 to <160**	**160 to <320**	**≥320**
**N**	130	512	93	214	120	35	50
**Dose discontinuations**[a]							
Incidence, n (%)	3 (2.3%)	23 (4.5%)	4 (4.3%)	6 (2.8%)	11 (9.2%)	1 (2.9%)	1 (2.0%)
**Local cutaneous reactions at the injection site**[b]							
Incidence, n (%)	0 (0%)	65 (12.7%)	8 (8.6%)	28 (13.1%)	18 (15.0%)	8 (22.9%)	3 (6.0%)
% per injection							
Mean (SD)	0.0 (0.0)	2.2 (8.2)	0.8 (2.8)	2.8 (10.1)	2.2 (8.1)	3.8 (8.4)	1.3 (5.6)
Median (Q1, Q3)	0.0 (0.0, 0.0)	0.0 (0.0, 0.0)	0.0 (0.0, 0.0)	0.0 (0.0, 0.0)	0.0 (0.0, 0.0)	0.0 (0.0, 0.0)	0.0 (0.0, 0.0)
**Flu-like reactions**[c]							
Incidence, n (%)	0	7 (1.4%)	0	6 (2.8%)	0	1 (2.9%)	0
% per injection							
Mean (SD)	0.0 (0.0)	0.3 (3.5)	0.0 (0.0)	0.5 (3.7)	0.0 (0.0)	1.7 (9.9)	0.0 (0.0)
Median (Q1, Q3)	0.0 (0.0, 0.0)	0.0 (0.0, 0.0)	0.0 (0.0, 0.0)	0.0 (0.0, 0.0)	0.0 (0.0, 0.0)	0.0 (0.0, 0.0)	0.0 (0.0, 0.0)

[a]Discontinuation of treatment due to adverse events.
[b]Local cutaneous reactions at the injection site were defined as injection site erythema, injection site swelling, injection site pruritus, injection site pain which started the day of subcutaneous injection and persisted (start to stop) for 2 days or more.
[c]Flu-like reactions were defined as either (a) influenza-like illness or (b) pyrexia or feeling hot or body temperature increased, plus at least two of the following: chills, myalgia, and arthralgia, started on day of injection or the next day.
ASO, antisense oligonucleotide; SD, standard deviation.
