# Supplementary material for: Integrated Assessment of Phase 2 Data on GalNAc3-Conjugated 2′-O-Methoxyethyl-Modified Antisense Oligonucleotides
Source: Nucleic Acid Ther. 2023 Feb 1;33(1):72–80. doi: 10.1089/nat.2022.0044 (PMC10623620; doi:10.1089/nat.2022.0044)
Supplement: Supplemental data [file Suppl_TableS18.pdf]

Supplemental Table 18. Tolerability results on total study population and by dose regimen cohort

			Dose (mg/month)				
	**Placebo**	**Total ASO**	**>0 to <40**	**40 to <80**	**80 to <160**	**160 to <320**	**>=320**
**Total, N**	130	512	93	214	120	35	50
**LCRIS*, n (%)**							
Total	0 (0%)	65 (12.7%)	8 (8.6%)	28 (13.1%)	18 (15.0%)	8 (22.9%)	3 (6.0%)
Monthly	0 (0%)	24 (9.5%)	6 (8.6%)	16 (11.2%)	2 (5.0%)		
Weekly	0 (0%)	41 (15.8%)	2 (8.7%)	12 (16.9%)	16 (20%)	8 (22.9%)	3 (6.0%)
**LCRIS % Inj, Mean (SD)**							
Total	0.0 (0.0)	2.2 (8.2)	0.8 (2.8)	2.8 (10.1)	2.2 (8.1)	3.8 (8.4)	1.3 (5.6)
Monthly	0 (0.0)	1.5 (5.4)	0.9 (3.0)	1.9 (6.1)	1.3 (5.8)		
Weekly	0 (0.0)	2.9 (10.3)	0.5 (1.8)	4.6 (15.2)	2.7 (9.1)	3.8 (8.4)	1.3 (5.6)
**LCRISv2†, n (%)**							
Total	0	8 (1.6%)	0	3 (1.4%)	4 (3.3%)	0	1 (2.0%)
Monthly	0	3 (1.2%)	0	2 (1.4%)	1 (2.5%)		
Weekly	0 (0%)	5 (1.9%)	0 (0%)	1 (1.4%)	3 (3.8%)	0 (0%)	1 (2.0%)
**LCRISv2 % Inj, Mean (SD)**							
Total	0.0 (0.0)	0.1 (1.3)	0.0 (0.0)	0.1 (1.5)	0.1 (0.91)	0.0 (0.0)	0.3 (2.4)
Monthly	0.0 (0.0)	0.0 (0.63)	0.0 (0.0)	0.1 (0.8)	0.0 (0.0)		
Weekly	0 (0.0)	0.2 (1.7)	0 (0.0)	0.3 (2.3)	0.1 (1.1)	0 (0.0)	0.3 (2.4)
**FLR‡, n (%)**							
Total	0	7 (1.4%)	0	6 (2.8%)	0	1 (2.9%)	0
Monthly	0	5 (2.0%)	0	5 (3.5%)	0		
Weekly	0 (0%)	2 (0.8%)	0 (0%)	1 (1.4%)	0 (0%)	1 (2.9%)	0 (0%)
**FLR, % Injections, Mean (SD)**							
Total	0.0 (0.0)	0.3 (3.5)	0.0 (0.0)	0.5 (3.7)	0.0 (0.0)	1.7 (9.9)	0.0 (0.0)
Monthly	0.0 (0.0)	0.4 (3.44)	0.0 (0.0)	0.7 (4.56)	0.0 (0.0)		
Weekly	0 (0.0)	0.2 (3.6)	0 (0.0)	0.0 (0.2)	0 (0.0)	1.7 (9.9)	0 (0.0)

	Placebo	Total ASO	Dose (mg/month) >0 to <40	40 to <80	80 to <160	160 to <320	>=320
**Dose Discontinuations, n (%)**							
Total	3 (2.3%)	23 (4.5%)	4 (4.3%)	6 (2.8%)	11 (9.2%)	1 (2.9%)	1 (2.0%)
Monthly	3 (4.6%)	9 (3.6%)	3 (4.3%)	4 (2.8%)	2 (5.0%)		
Weekly	0	14 (5.4%)	1 (4.3%)	2 (2.8%)	9 (11.3%)	1 (2.9%)	1 (2.0%)

* Local cutaneous reactions at the injection site (LCRIS) were defined as injection site erythema, injection site swelling, injection site pruritus, injection site pain which started the day of SC injection, and persisted (start to stop) for 2 days or more.

† Local cutaneous reaction at injection site v2 (LCRISv2) is defined as (A) moderate or severe Injection site erythema, Injection site swelling, Injection site pruritus, Injection site pain that started on the day of injection, persisted for at least two days; or (B) any AE at the injection site, regardless of severity, that leads to discontinuation of study drug, where AE at the injection site is the principal reason for discontinuation.

‡ Flu-like reaction (FLR) were defined as either (A) Influenza like illness or (B) Pyrexia or feeling hot or body temperature increased, plus at least two of the following: Chills, Myalgia, and Arthralgia, started on day of injection or the next day. All events were reported as Influenza-like illness.
